# Long-term suboptimal dietary trace element supply does not affect trace element homeostasis in murine cerebellum

**DOI:** 10.1093/mtomcs/mfae003

**Published:** 2024-01-31

**Authors:** Sharleen Friese, Giovanna Ranzini, Max Tuchtenhagen, Kristina Lossow, Barbara Hertel, Gabriele Pohl, Franziska Ebert, Julia Bornhorst, Anna Patricia Kipp, Tanja Schwerdtle

**Affiliations:** Department of Food Chemistry, Institute of Nutritional Science, University of Potsdam, Arthur-Scheunert-Allee 114-116, 14558 Nuthetal, Germany; TraceAge—DFG Research Unit on Interactions of Essential Trace Elements in Healthy and Diseased Elderly (FOR 2558), Berlin-Potsdam-Jena-Wuppertal, Germany; Department of Food Chemistry, Institute of Nutritional Science, University of Potsdam, Arthur-Scheunert-Allee 114-116, 14558 Nuthetal, Germany; Department of Food Chemistry, Institute of Nutritional Science, University of Potsdam, Arthur-Scheunert-Allee 114-116, 14558 Nuthetal, Germany; TraceAge—DFG Research Unit on Interactions of Essential Trace Elements in Healthy and Diseased Elderly (FOR 2558), Berlin-Potsdam-Jena-Wuppertal, Germany; TraceAge—DFG Research Unit on Interactions of Essential Trace Elements in Healthy and Diseased Elderly (FOR 2558), Berlin-Potsdam-Jena-Wuppertal, Germany; Nutritional Physiology, Institute of Nutritional Sciences, Friedrich Schiller University Jena, Dornburger Str. 24, 07743 Jena, Germany; Department of Food Chemistry, Institute of Nutritional Science, University of Potsdam, Arthur-Scheunert-Allee 114-116, 14558 Nuthetal, Germany; Department of Food Chemistry, Institute of Nutritional Science, University of Potsdam, Arthur-Scheunert-Allee 114-116, 14558 Nuthetal, Germany; Department of Food Chemistry, Institute of Nutritional Science, University of Potsdam, Arthur-Scheunert-Allee 114-116, 14558 Nuthetal, Germany; TraceAge—DFG Research Unit on Interactions of Essential Trace Elements in Healthy and Diseased Elderly (FOR 2558), Berlin-Potsdam-Jena-Wuppertal, Germany; Food Chemistry, Faculty of Mathematics and Natural Sciences, University of Wuppertal, Gaußstraße 20, 42119 Wuppertal, Germany; TraceAge—DFG Research Unit on Interactions of Essential Trace Elements in Healthy and Diseased Elderly (FOR 2558), Berlin-Potsdam-Jena-Wuppertal, Germany; Nutritional Physiology, Institute of Nutritional Sciences, Friedrich Schiller University Jena, Dornburger Str. 24, 07743 Jena, Germany; Department of Food Chemistry, Institute of Nutritional Science, University of Potsdam, Arthur-Scheunert-Allee 114-116, 14558 Nuthetal, Germany; TraceAge—DFG Research Unit on Interactions of Essential Trace Elements in Healthy and Diseased Elderly (FOR 2558), Berlin-Potsdam-Jena-Wuppertal, Germany; German Federal Institute for Risk Assessment (BfR), Max-Dohrn-Str. 8-10, 10589 Berlin, Germany

**Keywords:** ageing, cerebellum, genomic stability, sex, trace element homeostasis, trace element transporter

## Abstract

The ageing process is associated with alterations of systemic trace element (TE) homeostasis increasing the risk, e.g. neurodegenerative diseases. Here, the impact of long-term modulation of dietary intake of copper, iron, selenium, and zinc was investigated in murine cerebellum. Four- and 40-wk-old mice of both sexes were supplied with different amounts of those TEs for 26 wk. In an adequate supply group, TE concentrations were in accordance with recommendations for laboratory mice while suboptimally supplied animals received only limited amounts of copper, iron, selenium, and zinc. An additional age-adjusted group was fed selenium and zinc in amounts exceeding recommendations. Cerebellar TE concentrations were measured by inductively coupled plasma–tandem mass spectrometry. Furthermore, the expression of genes involved in TE transport, DNA damage response, and DNA repair as well as selected markers of genomic stability [8-oxoguanine, incision efficiency toward 8-oxoguanine, 5-hydroxyuracil, and apurinic/apyrimidinic sites and global DNA (hydroxy)methylation] were analysed. Ageing resulted in a mild increase of iron and copper concentrations in the cerebellum, which was most pronounced in the suboptimally supplied groups. Thus, TE changes in the cerebellum were predominantly driven by age and less by nutritional intervention. Interestingly, deviation from adequate TE supply resulted in higher manganese concentrations of female mice even though the manganese supply itself was not modulated. Parameters of genomic stability were neither affected by age, sex, nor diet. Overall, this study revealed that suboptimal dietary TE supply does not substantially affect TE homeostasis in the murine cerebellum.

## Graphical Abstract

**Graphical Abstract:**
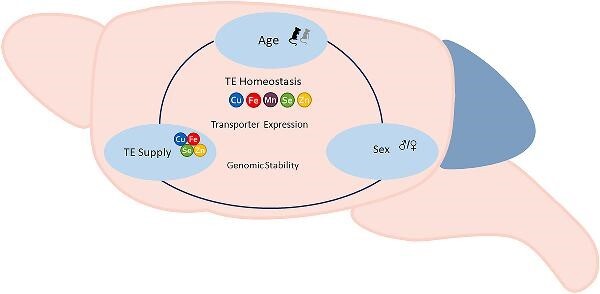
Ageing is associated with an altered TE status. In the cerebellum of mice, TE homeostasis was most strongly influenced by age whereas both suboptimal and age-adjusted dietary TE supply did not have substantial impact. Parameters of genomic stability were neither affected by age, sex nor diet. This emphasizes the limitations in counteracting age- and sex-specific changes of TE concentrations in the murine brain via dietary supply.

## Introduction

Trace elements (TEs) are essential micronutrients with indispensable roles in a wide range of cellular processes involved in metabolism, the redox, and immune system. An imbalance in TE concentrations, such as deficiency as well as excess, has been linked to a variety of diseases.^1,2^ While appropriate dietary intake of TEs represents a relevant aspect of human health, the TE status in the body is further influenced by sex and age.^3^ Imbalanced TE homeostasis is associated with variations in cellular redox status, which has been shown to be one of the key players in the ageing process by affecting genomic stability. Consequently, an age-dependent increase in oxidative stress is considered to lead to accumulation of DNA damage while at the same time stress response and DNA repair capacity decline.^4–6^

Age-related shifts in serum TE concentrations, such as decreases of manganese (Mn), selenium (Se), and zinc (Zn) as well as increased concentrations of copper (Cu), iron (Fe), and iodine (I) have already been detected in humans.^3^ In mice, serum concentrations of Cu and Se have been observed to be affected by age.^7,8^ For both TEs as well as for the Se transporter selenoprotein P, serum levels were increased in aged mice while hepatic concentrations of Cu and Se were reduced. Since the liver represents the main organ for TE distribution, this potentially points to a higher transport rate to peripheral organs, including the brain. Correspondingly, there is evidence for an age-associated weakening of the blood–brain barrier, which may promote an accumulation of TEs within the brain.^9^ Additionally, the Fe status markers serum transferrin and hepatic Fe were found to be increased in old, especially female mice.^8^

This study aimed to analyse TE concentrations in the brain in comparison to the observed age-related changes in serum and liver. In contrast to the high restorative potential of the liver, cerebral neurons as post-mitotic cells are limited in this respect. Accordingly, they are supposed to be more vulnerable to changes in TE concentrations and subsequent cellular alterations. Ageing is strongly associated with a decline of motor and cognitive performance, which can be partially attributed to altered TE homeostasis in the brain.^10^ Loss of cerebellar neurons manifesting in a lower cerebellar volume is not only observed during ageing but also in Alzheimer's disease correlating with progression and severity.^11–14^ In the cerebellum of Alzheimer's disease patients, concentrations of Cu, Fe, Mn, Se, and Zn were found to be decreased in comparison to healthy controls whereas in Parkinson's and Huntington's disease, TE imbalances in cerebellum are rarely observed.^15^

Age- and disease-related neuronal changes are mainly driven by oxidative stress. The murine cerebellum contains around 59% of all brain neurons.^16^ Cerebellar neurons are particularly vulnerable to oxidative stress due to intrinsic characteristics including lower ATP levels than cortical neurons but also distinct gene expression patterns regarding oxidative stress and inflammatory response.^17^ Moreover, compared to other organs, neuronal antioxidant system capacities are limited due to low concentrations of antioxidant enzymes as well as low molecular weight antioxidants.^18^ TEs such as Mn, Se, and Zn are important players in antioxidative systems as essential constituents of antioxidant enzymes.^19,20^ Thus, lower levels of those TEs could also result in elevated oxidative stress. This may influence a number of cellular macromolecules, including DNA, either directly resulting in oxidative DNA damage or indirectly by affecting other molecules involved for example in preservation of DNA integrity.

Therefore, this study aimed to quantify TE concentrations and genomic stability in the murine cerebellum. For this purpose, 4- and 40-wk-old mice of both sexes were supplied with suboptimal or adequate amounts of selected TEs including Cu, Fe, Se, and Zn to analyse how age, sex, and dietary TE supply influence cerebellar TE profiles. Additionally, aiming to counteract the age-dependent decrease of serum concentrations of Se and Zn observed in humans, a third diet group was fed age-adjusted amounts of Se and Zn via the drinking water, exceeding recommendations 4- and 6-fold, respectively.^3^ Furthermore, the mRNA expression of selected genes facilitating TE transport as well as markers of genomic stability were studied in the cerebellum.

## Experimental section

### Animal husbandry and dosage information (dietary intervention)

A total of 106 wild-type C57BL/6Jrj mice were obtained from Janvier (Saint-Berthevin, France). As one of the most commonly used mouse strains for basic research, this strain was chosen since precursory experiments were all carried out in the same mouse model so that all results can be adequately compared to previously gathered data. Polycarbonate cages were provided with limited environmental enrichment material to prevent uncontrolled TE intake as far as possible. The animals were housed on quotidian 12:12 h light/dark cycles at constant room temperature of 22°C and humidity of 55% with food and water *ad libitum*. At the age of 4 wk (after weaning) and 40 wk, mice were fed a diet with low concentrations of five selected TEs [(modified AIN93M, Cargill, Granovit, Kaiseraugst, Switzerland; 0.44 mg/kg feed Cu, 9.6 mg/kg feed Fe, 0.75 mg/kg feed Mn, 0.07 mg/kg feed Se and 4.7 mg/kg feed Zn, respectively, determined via inductively coupled plasma–tandem mass spectrometry (ICP–MS/MS)] but also low in I (0.1 mg/kg feed) and Mg (40 mg/kg feed) as reported before.^21^ To ensure adequate supply, drinking water for all mice was fortified with 0.05 mg/kg I (KI) and 500 mg/kg Mg (MgSO_4_).

**Fig. 1:**
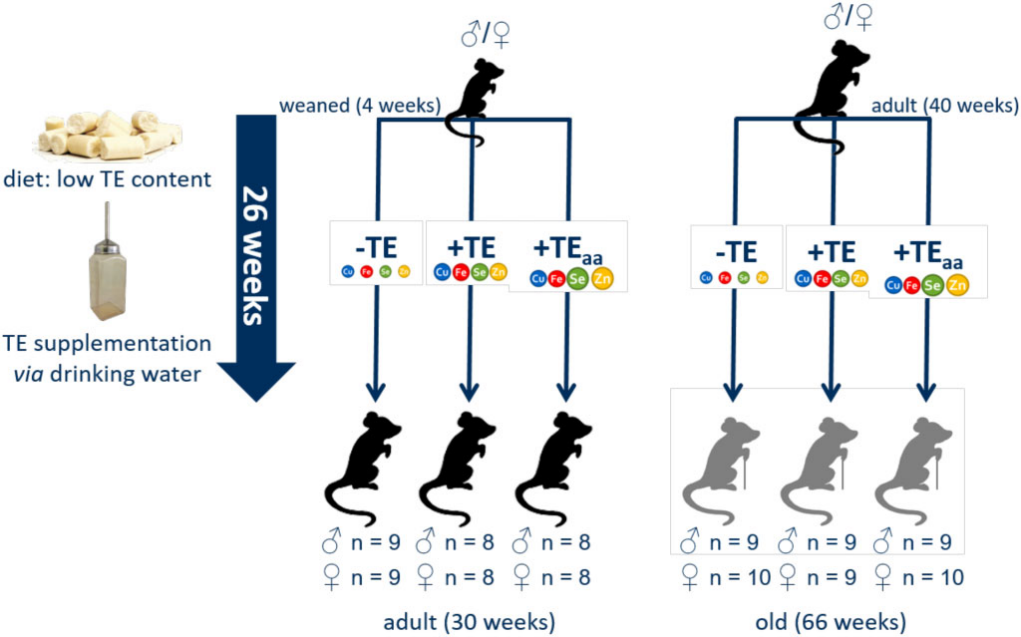
Study design of feeding regime. A total of 52 4-wk-old and 56 40-wk-old male and female C57BL/6Jrj mice received −TE, +TE, or +TE_aa_ diet for 26 wk. This was achieved by feeding a diet low in selected trace elements and adjusting supplied trace element levels via the drinking water.

The animals were divided into 12 groups, which received either suboptimal (−TE), adequate (+TE) or age-adjusted (+TE_aa_) supply of TEs. Here, the adequately supplied group served as control group. To reduce total animal numbers, it was decided not to create a further control group fed a standard chow diet, difficult to compare to other results based on drastic shifts of diet matrices. Each TE regiment was fed to four groups of adult and old male and female mice, respectively. Groups were assigned randomly after matching body weights. Those groups consisted of 8 to 10 animals each, housed individually (older animals) or up to three animals per cage. Initially, eight (adult) or ten animals (old) were allocated to each group, whereby more older animals were integrated to take into account possible age-related losses in the course of the experiment. All animals that arrived healthy participated in the experiment without any further inclusion criteria set. This resulted in variances in group size, which are stated in detail in Supplementary Table S1 in compliance with the Animal Research: Reporting of *In Vivo* Experiments guidelines.^22^ Furthermore, an overview of the feeding regime is depicted in Fig. 1.

Changes in dietary TE supply were achieved by varying the amount of TEs of interest in filtered ultrapure water as previously published.^8^ In humans Mn deficiency not based on genetic defects is very rare; therefore, Mn concentrations were not altered between the different groups, all receiving 9.25 mg/kg feed and water intake (in the following referred to as feed) Mn (MnCl_2_).^23^ For mice in the −TE group no other TEs were administered additionally. Animals fed the +TE diet were supplied with 5.56 mg/kg feed Cu (CuSO_4_), 25.4 mg/kg feed Fe (FeCl_2_), 0.08 mg/kg feed Se (Na_2_SeO_3_) and 25.3 mg/kg feed Zn (ZnSO_4_), according to feeding recommendations for adequate supply for laboratory mice.^24^ The +TE_aa_ group received the same amount of Cu and Fe as animals of the +TE group but higher concentrations of Se and Zn with 0.53 mg/kg feed and 175.3 mg/kg feed, respectively. Total TE concentrations of the different diets in food and drinking water are listed in Table 1. Since TE supply may influence feed and water intake, both were monitored for each cage. In order to maintain the TE supply levels aimed for, TE concentrations in drinking water were adjusted weekly to each group's respective intake. This constant monitoring process highlights the reason for choosing to supplement TEs via the drinking water as customized diets cannot be adjusted and produced fast enough to that extent. Due to this adjustment procedure, group allocations were not blinded during the feeding period but only after tissue harvesting.

**Table 1.** TE supply of mice^a^

	Total supply [mg kg^−1^]			Fold changes		
TE	−TE	+TE	+TE_aa_	−TE vs. +TE	−TE vs. +TE_aa_	+TE_aa_ vs. +TE
Cu	0.44	6.00	6.00	0.07	0.07	0
Fe	9.62	35.0	35.0	0.3	0.3	0
Mn	10.0	10.0	10.0	0	0	0
Se	0.07	0.15	0.60	0.5	0.12	4
Zn	4.75	30.0	180	0.15	0.03	6

^a^Total content of the trace elements copper (Cu), iron (Fe), manganese (Mn), selenium (Se), and zinc (Zn) in feed and water within the animal experiment as previously published.^8^

After 26 wk, at the age of 30 (adult) and 66 wk (old), mice were anesthetized with isoflurane (Cp-pharma, Burgdorf, Germany) followed by dissection of the cerebellum. Samples were immediately snap-frozen in liquid nitrogen and stored at −80°C.

All animal procedures were approved by and performed according to national guidelines of the Ministry of Environment, Health and Consumer Protection of the federal state of Brandenburg, Germany (2347-44-2017) and institutional guidelines of the German Institute of Human Nutrition Potsdam-Rehbruecke, Germany.

### ICP–MS/MS analysis of TEs

For analysis of TE concentrations via ICP–MS/MS, 30 mg of cerebellar tissue were subjected to microwave-assisted acid digestion as described before.^25^ Briefly, HNO_3_ (65% Suprapur, Merck, Darmstadt, Germany) and H_2_O_2_ (30%, Merck) were used for microwave-assisted digestion using polytetrafluoroethylene microwave vessels and internal as well as isotope-dilution standards for Se measurement via isotope dilution analysis. After digestion, cerebellum samples were diluted 1:5 to a final concentration of HNO_3_ of 4.5% (v/v) and analysed via ICP–MS/MS (8800 ICP–QQQ–MS, Agilent Technologies, Waldbronn, Germany) as addressed in detail by Schwarz and colleagues.^25^ Quality of results was assured by certified reference materials ERM-BB 422 (fish muscle) and ERM-BB 186 (pig kidney, both Merck) analysed in parallel with the samples according to the described protocol.

### RNA isolation, reverse transcription, and RT-qPCR

Total cerebellar RNA was isolated from the same mouse study using TRIzol Reagent [Thermo Fisher Scientific (Invitrogen), Waltham, USA] according to the manufacturer's instructions. Detailed information on nucleic acid extraction according to MIQE Guidelines can be found in the supplementary information.^26^ Reverse transcription and quantitative real-time PCR (RT-qPCR) were conducted as described previously.^8^ In short, DNase I (Thermo Fisher Scientific) was used according to manufacturer's instructions to eliminate genomic DNA prior to reverse transcription of 4 µg RNA (qScript cDNA synthesis, Quanta BioSciences, Beverly, USA). Generated complementary DNA (cDNA) was amplified by PerfeCTa SYBR Green Supermix (Quanta Biosciences). Priming oligonucleotide sequences and RT-qPCR target information are listed in Supplementary Table S2 according to MIQE Guidelines.^26^ RT-qPCR was conducted in the CFX Connect Real-time PCR Detection System (Bio-Rad Laboratories, Munich, Germany). Standard curves were used to correct for variations in PCR efficiencies and calculate copy numbers. Finally, gene expression levels were normalized to a composite factor based on the reference genes glyceraldehyde-3-phosphate dehydrogenase (*Gapdh*) and ribosomal protein L13A (*Rpl13a*). Only a subset of selected genes directly related to the observed changes in cerebellar TE concentrations will be discussed in the part results and discussion. For more detailed information on this section see Supplementary Information: RNA isolation, reverse transcription, and qPCR.

### Base excision repair incision activity assay

Base excision repair (BER) incision activity toward oligonucleotides containing 8-oxoguanine (8-oxo-dG), 5-hydroxyuracil (5-OH-dU) or an apurinic/apyrimidinic (AP) site analogue was determined as described before.^27^ Briefly, tissue extracts were prepared from 30 mg snap-frozen cerebellum tissue and incubated with fluorescently labeled hairpin-structured oligonucleotides containing one of the three different DNA lesions investigated. Intact and incised oligonucleotides were separated by denaturing PAGE and quantified via fluorescent Cy5-labeling using a Chemidoc MP imaging system and appropriate Image Lab software (Bio-Rad Laboratories). Incision activity can be deduced from the ratio of incised to intact oligonucleotide.

### Alkaline comet assay

The method of choice for detection of DNA strand breaks and alkali-labile sites is the alkaline comet assay according to OECD guideline 489 reported according to the recommendations for minimum information for reporting on the comet assay (MIRCA).^28,29^ However, due to the sensitivity of brain tissue to storage time the alkaline comet assay was no longer possible to perform quantitatively in the present tissue. This was confirmed in a storage stability experiment performed in fresh cerebellum and tissue frozen for 1 wk as well as 1, 3, 6, and 12 mo as described previously (Supplementary Fig. S3).^27^ In brief, cells were isolated and embedded in agarose gel before being subjected to DNA unwinding and electrophoresis in alkaline solution. Stained comet heads and tails were analysed by semi-automated image analysis software (Comet IV, Perceptive Instruments, Stone, UK) in 100 randomly selected cells per replicate.

### ELISA-based analysis of oxidative DNA damage

Relative 8-oxo-dG levels as common biomarker for oxidative DNA damage were determined as previously described.^8^ In short, DNA was isolated from 15 mg snap-frozen cerebellum using Qiagen DNeasy Kit (Qiagen, Hilden, Germany) and digested by 1 U benzonase (Sigma-Aldrich, St. Louis, USA) and 1 U alkaline phosphatase (Thermo Fisher Scientific) at 37°C. A total of 5 µg of digested DNA was employed in a DNA/RNA Oxidative Damage ELISA Kit (Cayman Chemicals, Ann Arbor, USA) and prepared according to the manufacturer's instructions. Absorbance measurement was performed after 90 min at 405 nm using an Infinite 200 Pro microplate reader (Tecan, Männedorf, Switzerland). Absolute quantification was based on external standard curves while for relative assessment variances were compared to mean +TE adult male 8-oxo-dG concentration.

### Measurement of global DNA (hydroxy)methylation via HPLC–MS/MS

As described before, DNA was extracted from frozen cerebellum tissue via phenol/chloroform/isoamylalcohol extraction followed by enzymatic hydrolysis to obtain respective nucleosides by use of micrococcal nuclease from *Staphylococcus aureus*, bovine spleen phosphodiesterase and alkaline phosphatase (all Sigma-Aldrich).^21^ Quantification of methylated (mdC) and hydroxymethylated (hmdC) cytidine in total DNA was conducted on high pressure liquid chromatography (HPLC)–MS/MS (HPLC: Agilent 1260 Infinity, MS/MS: Agilent 6495A).

### Statistical analysis

Statistics were performed in GraphPad Prism 8 (GraphPad Software, La Jolla, USA). In accordance with precursory experiments, statistical outliers were removed by Grubb's test. This test was chosen to minimize potential bias because it only excludes one data point per group. Normal distribution and homogeneity of variances were tested by Shapiro–Wilk and Brown–Forsythe test. Three-way analysis of variance (ANOVA) for the independent variables age, sex and TE supply was followed by Bonferroni *post hoc* test. See Table 2 and Supplementary Table S3 for an overview of mean values ± SD or SEM, respectively, as well as *P*-values for all parameters analysed in this study. For correlation of element concentrations and gene expression data Spearman’s correlation was performed with correlation coefficients displayed as heatmaps in Supplementary Fig. S2. As the type I error was set to 0.05, statistical significances are indicated as *P < 0.05, **P < 0.01, ***P < 0.001.

**Table 2.** Overview of mean values and statistical testing of the different diets for TEs and selected markers in murine cerebellum^a^

		Diet			Statistics		
Parameter	Unit	−TE	+TE	+TE_aa_	−TE vs. +TE	−TE vs. +TE_aa_	+TE_aa_ vs. +TE
Cu	[mg kg^−1^]	4.71 ± 0.19	5.38 ± 0.15	4.98 ± 0.15	**0.0018**	0.2245	0.0536
Fe	[mg kg^−1^]	17.98 ± 0.39	18.75 ± 0.29	18.63 ± 0.33	0.1010	0.2218	0.7175
Mn	[mg kg^−1^]	0.42 ± 0.01	0.34 ± 0.01	0.39 ± 0.01	**0.0001**	**0.0221**	**0.0001**
Se	[mg kg^−1^]	0.173 ± 0.004	0.178 ± 0.004	0.181 ± 0.005	0.3073	0.2136	0.8034
Zn	[mg kg^−1^]	10.08 ± 0.23	10.47 ± 0.21	11.07 ± 0.36	0.2164	**0.0224**	0.1627
rel. *Cp* mRNA		1.10 ± 0.29	0.99 ± 0.15	1.36 ± 0.60	0.1430	**0.0402**	**0.0065**
rel. *Hamp* mRNA		1.01 ± 0.19	1.08 ± 0.34	0.94 ± 0.14	0.6584	0.0590	**0.0253**
rel. *Lrp2* mRNA		1.21 ± 0.43	1.15 ± 0.43	0.97 ± 0.32	0.6594	**0.0169**	0.0983
rel. *Slc39a14* mRNA		1.23 ± 0.29	1.14 ± 0.36	1.14 ± 0.34	0.3006	**0.0261**	0.2192
rel. *Slc30a1* mRNA		1.16 ± 0.20	1.12 ± 0.21	1.07 ± 0.19	0.3715	**0.0202**	0.1964
rel. *Slc30a6* mRNA		1.01 ± 0.20	0.96 ± 0.17	0.91 ± 0.13	0.3195	**0.0193**	0.1772
rel. 8-oxo-dG level		0.89 ± 0.04	0.97 ± 0.04	1.01 ± 0.04	0.2652	**0.0160**	0.2349

^a^Values shown as mean ± SEM or SD (gene expression data) and *P*-values for statistical analysis of adult (30 wk) and old (66 wk) male and female C57BL/6Jrj mice receiving **−**TE, +TE, or +TE_aa_ diet for 26 wk. Statistical testing based on three-way ANOVA and Bonferroni *post hoc* test. *P*-values < 0.05 are indicated in bold/underlined.

## Results and discussion

Adult (30 wk) and old mice (66 wk) were analysed after receiving feed containing suboptimal (−TE), adequate (+TE), or age-adjusted (+TE_aa_) TE concentrations for 26 wk. Previously, TE concentrations have been analysed in serum and liver of these mice, revealing diet-induced changes of Se, Cu, and Fe concentrations.^8^ Even though dietary Zn supply was modulated, no changes in serum or hepatic Zn concentrations were observed. Based on this, the concentrations of Cu, Fe, Mn, Se, and Zn were also analysed in the cerebellum of these mice as one peripheral organ depending on circulating TE concentrations.

### Cerebellar weight was affected by age

First, the weight of the cerebellum was assessed. Adult as well as old female mice displayed a higher relative cerebellar weight compared to male animals (Supplementary Fig. S1). This is in contrast to results from Airey and colleagues, which could show that C57BL/6 J mice do not display a sexual dimorphism of the cerebellar weight.^30^ However, the average age of these mice was only 11 and 14 wk, so the discrepancies might be due to the age differences in both studies. Furthermore, as differences in cerebellar anatomy exist between the sexes as shown by Spring *et al.*, possible changes in the cerebellar weight might occur at an older age.^31^ The decline of the cerebellar weight in relation to age was also more pronounced in female animals, especially in the −TE group (Supplementary Fig. S1 and Table S3). The overall shrinkage of the cerebellum with age is widely known.^11–13^ Since there is evidence for a greater age-related decline of the hippocampus in female mice, it is possible that the same occurs on the cerebellar level.^32,33^

### Concentrations of cerebellar Cu and Fe were elevated in relation to age

**Fig. 2:**
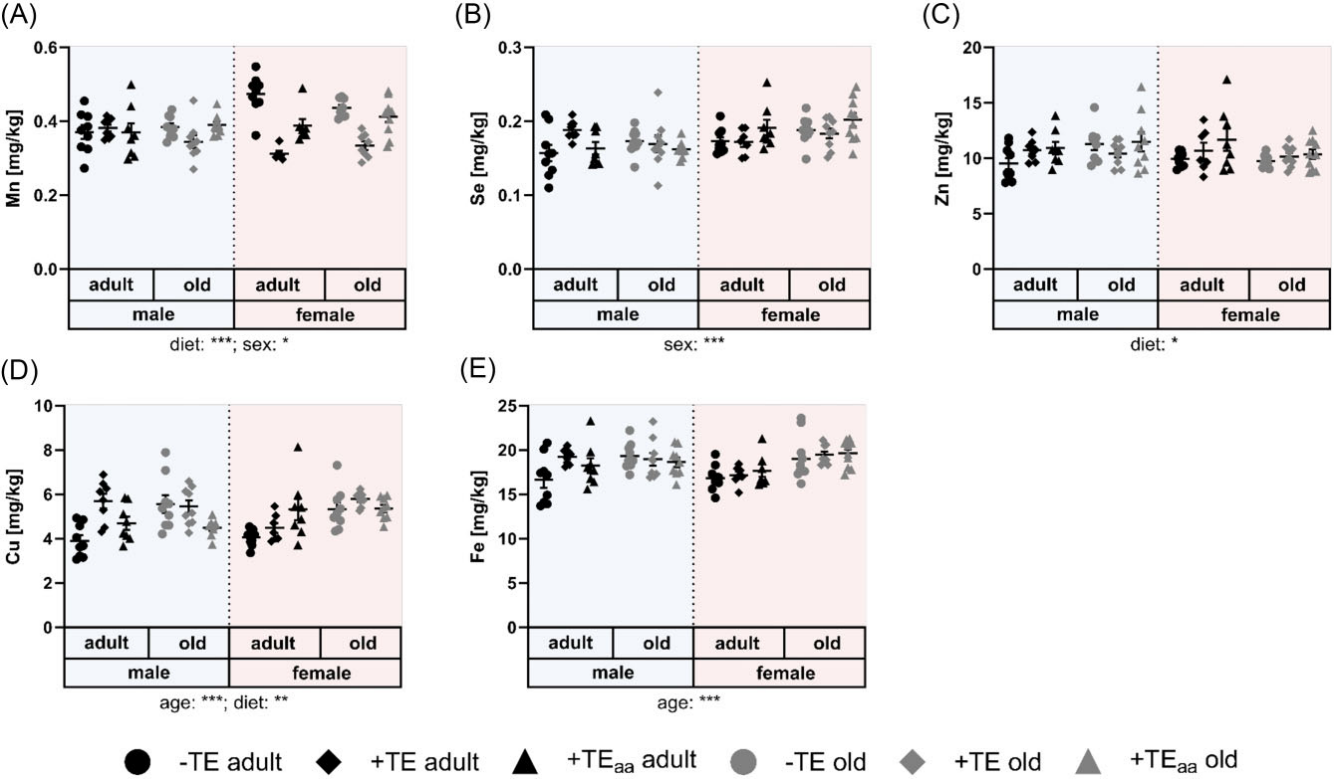
TE content in murine cerebellum. Concentrations of Mn (A), Se (B), Zn (C), Cu (D), and Fe (E) in cerebellum of adult (30 wk) and old (66 wk) male and female C57BL/6Jrj mice receiving −TE, +TE, or +TE_aa_ diet for 26 wk determined via ICP–MS/MS. Data points shown with mean ± SEM. Statistical testing based on three-way ANOVA and Bonferroni *post hoc* test with *P < 0.05, **P < 0.01, ***P < 0.001. Detailed results of statistical testing summarized in Supplementary Table S3.

Irrespective of TE supply and sex, no age-related changes of Mn, Se, and Zn concentrations were observed in the cerebellum (Fig. 2A–C), even though hepatic and serum Se concentrations of these mice increased with age.^8^ In contrast, cerebellar Cu and Fe concentrations were elevated in old mice (Fig. 2D, E). However, the age-related increase of Cu was only observed in the −TE groups (fold change (FC) of 1.4, *P* < 0.01 in males and FC of 1.3, *P *= 0.08 in females, Table 1) while there was no effect in the other groups with adequate or age-adjusted TE supply. In parallel, serum Cu levels were also increased in old mice, again showing most pronounced effects in the −TE groups.^8^ Also for Fe, a tendency to increase was only observed in the −TE groups, especially in males, with FCs ranging from 1.13 to 1.16 (Supplementary Table S3) being related to higher serum transferrin concentrations in the −TE groups.^8^ Such a mild age-dependent increase of both elements has already been shown in the cerebellum of mice by colleagues before, ranging from FCs of 1.3 for Cu to 1.2 for Fe.^7^ A reason for more stable age-related effects might be the older age of the mice in this previous study. Also, for whole brain an increase in Fe concentrations was observed before, which was recently confirmed for both Cu and Fe using a metallomics approach in murine whole brain.^34–36^ Another study analysed TE concentrations during the whole life span of mice ranging from one to 104 wk of age. Considering only the age range studied herein, almost no or only a very minor increase of Fe and Cu in whole brain tissue was observed.^35^ Based on this, we can conclude that there was almost no age-dependent change of TE concentrations in cerebellum and an increase in Cu and Fe levels only became detectable under −TE conditions. The higher concentrations of Cu and Fe may also explain the greater decline of the cerebellar weight observed in the −TE group (Supplementary Fig. S1 and Table S3). Accordingly, comparison to other studies is limited by the fact that in most experiments mice are fed a standard chow diet in which TE concentrations exceed requirements.^7^

Especially in the nervous system an accumulation of Fe with age is of concern due to its implications in several neurodegenerative diseases. Lately, ferroptosis is discussed as contributing factor to their onset and progression.^37^ Consequently, in future studies assessment of ferroptosis markers via Fe speciation analysis or monitoring lipid peroxidation could provide valuable information. This is especially true in the context of oxidative stress, as ferroptosis for example includes reduction of glutathione synthesis as well as suppression of glutathione peroxidase 4, both vital players of the antioxidant system.^37^ Therefore, inhibition of ferroptotic processes has already shown promising results for treatment of, e.g. Alzheimer's, Parkinson's, and Huntington's disease as well as amyotrophic lateral sclerosis.^37,38^

**Fig. 3:**
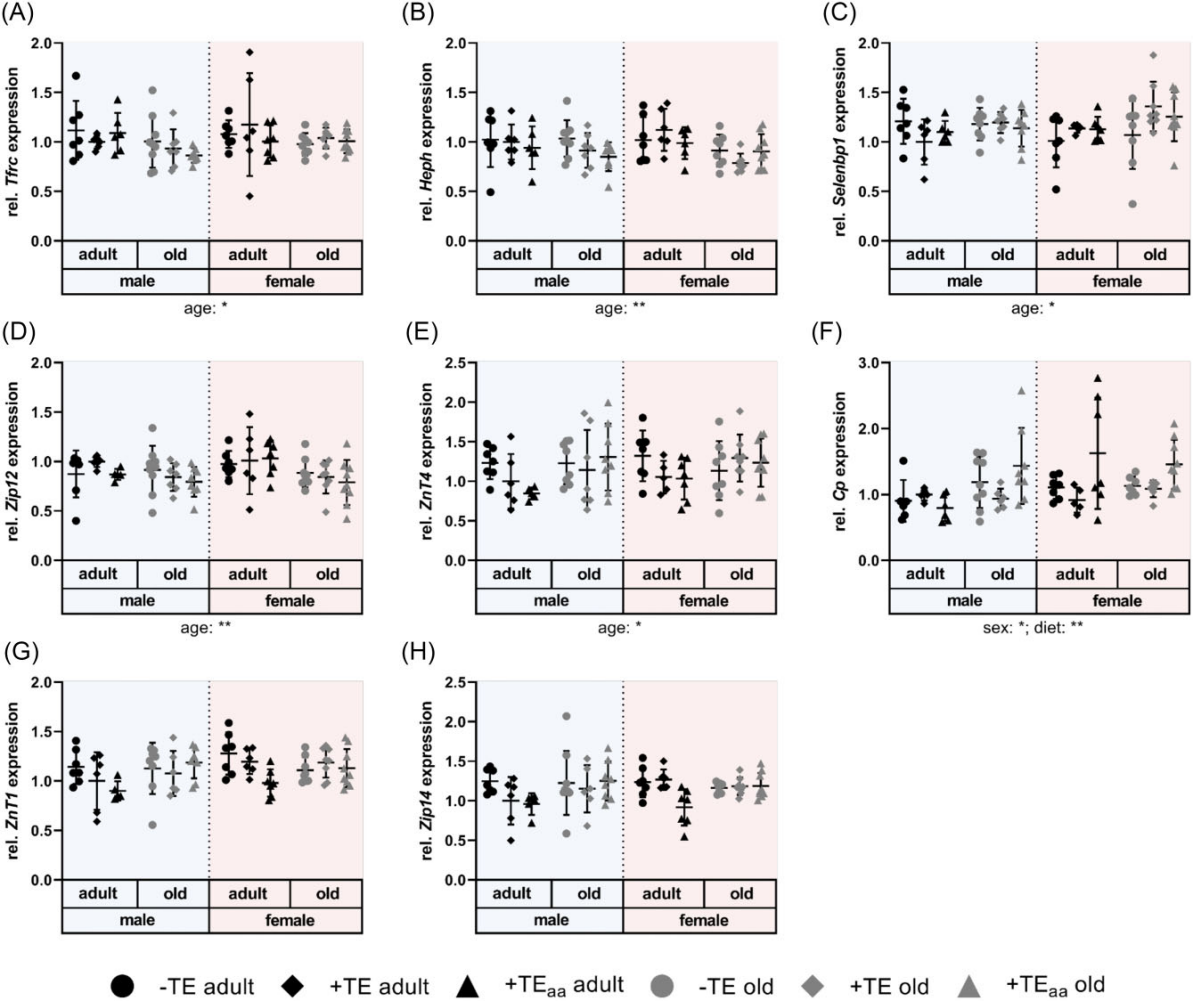
Cerebellar expression levels of TE-associated transporters. Gene expression levels examined in cerebellum of adult (30 wk) and old (66 wk) male and female C57BL/6Jrj mice receiving −TE, +TE, or +TE_aa_ diet for 26 wk determined via RT-qPCR analysis. Transcription levels of genes involved in Cu, Fe, I, Mn, Se, and Zn transport, including *Tfrc* (A), *Heph* (B), *Selenbp1* (C), *Slc39a12* (*Zip12*) (D), *Slc30a4* (*ZnT4*) (E), *Cp* (F), *Slc30a1* (*ZnT1)* (G), and *Slc39a14* (*Zip14*) (H) normalized to a composite factor based on reference genes *Gapdh* and *Rpl13a* with variances expressed as fold changes compared to +TE male adults (mean +TE male adult = 1). Data points shown with mean ± SD. Statistical testing based on three-way ANOVA and Bonferroni *post hoc* test with *P < 0.05, **P < 0.01. Detailed results of statistical testing summarized in Supplementary Table S3.

In parallel to mildly increased iron concentrations in cerebellum, mRNA expression of Fe-regulated proteins such as hephaestin (*Heph*) and transferrin receptor (*Tfrc*) was significantly downregulated in old mice (Fig. 3A, B). It is known that *Tfrc* is post-transcriptionally upregulated during Fe deficiency via the iron-responsive element–iron regulatory protein (IRE–IRP) signaling pathway, hence mRNA stability is modulated by IRP binding.^39–41^ Consequently, Fe concentrations are inversely related to the expression of *Tfrc*. Accordingly, the decrease of *Tfrc* mRNA in old mice could be a consequence of elevated cerebellar Fe concentrations observed in this study. However, there was no significant correlation observed between cerebellar Fe concentrations and *Tfrc* expression, neither in all animals nor after age correction (Supplementary Fig. S2). *Heph* mRNA on the other hand, does not harbor an IRE and is therefore not under the control of the IRE–IRP pathway.^42^ In this study, *Heph* expression was also downregulated in old mice, which was more obvious in female than in male mice (Fig. 3B). In the brain *Heph* is widely expressed and acts as a ferroxidase to enhance cellular Fe efflux and loading onto transferrin.^43,44^ Age-dependent regulation of *Heph* mRNA expression has been observed in rat brain with highest expression levels detected at the age of 9 wk followed by a decline until week 28. However, there was no clear correlation between *Heph* expression and Fe supply of the rats, as different expression patterns were observed in different brain regions.^42^ Also in the present study, there was no significant correlation of *Heph* expression and Fe concentrations in the cerebellum (Supplementary Fig. S2). HEPH is not only involved in Fe homeostasis but is also a Cu-dependent protein.^45^ Still, this regulation does not occur on mRNA levels since no positive correlation with brain Cu concentrations could be observed (Supplementary Fig. S2). The same holds true for the Cu transporters ATPase Cu transporting α/β polypeptides (ATP7A and ATP7B), as well as solute carrier family 31, member 1 (CTR1) which were not modulated by age or dietary intervention (Supplementary Table S3). Another interesting Cu-dependent protein is a methanethiol oxidase, which has initially been named selenium-binding protein 1 (Selenbp1).^46^ In the cerebellum, *Selenbp1* mRNA expression was upregulated in old mice, mainly in females (Fig. 3C). If this was driven by higher Cu concentrations needs to be further investigated. Even though no correlation between *Selenbp1* expression and Cu concentrations was observed, there is indeed a positive correlation with Ceruloplasmin (*Cp*) expression levels, which is especially pronounced (*r *= 0.303) in old animals (Supplementary Fig. S2). In *C. elegans*, SELENBP1 is a pro-ageing factor and could explain detrimental effects of increased Cu levels in old mice.^47^

In addition to the regulation of *Tfrc, Heph*, and *Selenbp1*, two more genes were regulated by ageing, namely *Slc39a12* and *Slc30a4*, encoding Zrt- and Irt-like protein 12 (ZIP12) and zinc transporter 4 (ZnT4) with the former being downregulated in old mice (mainly female) and the latter being upregulated (Fig. 3D, E). Both are Zn transport proteins and obviously do not correlate with Zn concentrations in cerebellum that were not affected by age (Fig. 2C, Supplementary Fig. S2). ZIP12 has been described to regulate mitochondrial function, a process that is commonly disturbed in many neurodegenerative diseases.^48^ ZnT4 is known to increase in the cerebellum of patients with preclinical stage of Alzheimer's disease, which could fit to the observed age-related upregulation in mice.^49^

### Chronic changes of dietary TE supply did not modulate cerebellar TE concentrations

The dietary intervention with either suboptimal supply of Cu, Fe, Se, and Zn or an age-adapted intake with Se and Zn supplementation in comparison to adequate TE intake was applied chronically for 6 mo. Cu, Fe, Se, and Zn supply was reduced to different degrees in the −TE group (Table 1). Cu was reduced to 7% of an adequate intake followed by Zn, which was reduced to 15%. In comparison, the reduction of Fe (30%) and Se (50%) was rather mild. Thus, it was not surprising that dietary effects were detected for Cu and Zn concentrations in cerebellum. For Cu, a significant decrease was only found in adult male mice (−TE vs. +TE) while there was clearly no dietary effect in old mice (Fig. 2D). This decrease in suboptimally supplied adult male mice is also mirrored by Fe. Apart from reduced feed content, also the interaction of both elements might contribute to that observation. As cofactor of CP, a multicopper ferroxidase, Cu plays an essential role for Fe metabolism. Upon reduced Cu availability in the liver, less stable holo-CP is released into circulation.^50^ This results in diminished Fe oxidation, which is necessary for Fe transport by transferrin. One consequence of that mechanism might be reduced Fe concentrations in peripheral organs, including the cerebellum. To support this hypothesis, however, further analysis of serum CP and its saturation are needed.

For Zn, a very small overall dietary effect was observed, which was again only visible in adult and not in old mice (Fig. 2C). On gene expression level, the most prominent diet-induced effect (Table 2) was observed for *Cp* (Fig. 3F) which, however, was not related to Cu concentrations in cerebellum (Supplementary Fig. S2) and accordingly was not different between −TE and +TE groups. Instead, *Cp* expression was significantly increased in +TE_aa_ animals in comparison to the +TE groups (except for adult males). Since cerebellar content of neither Cu nor Fe was significantly altered by age-adjusted TE supply we do not have an explanation for this phenomenon. An astrocyte-specific *Cp* knockout in mice revealed that Fe concentrations decreased in the cerebral cortex and hippocampus of young (6 mo) and old (18 mo) mice resulting in downregulation of ferritin levels.^51^ However, herein *ferritin heavy polypeptide* 1 mRNA expression was unaffected by age or dietary intervention (Supplementary Table S3). Except for *Cp*, no significant diet-induced effect on TE-related gene expression was observed.

In the age-adjusted diet Se and Zn were supplied in amounts exceeding the recommendations four and six times, respectively (Table 1). This was not reflected by a significant effect on Se and Zn concentrations in the cerebellum (Fig. 2B, C, Table 2). In general, Se concentrations were slightly upregulated in the cerebellum of female in comparison to male mice, which was most pronounced in the old +TE_aa_ groups (Fig. 2B). In a previous study, TE concentrations were measured in several organs of male and female mice, including cerebellum, which also revealed higher Se concentrations in female mice.^7^ It is possible that the elevated supply of Zn is already counter-regulated at the intestinal level limiting its systemic bioavailability.^52^ Consequently, neither total nor free serum Zn were affected in +TE_aa_ groups.^8^ The mRNA levels of *Slc30a1*, encoding Zn exporter ZnT1, were downregulated in mice fed the age-adjusted diet (Fig. 3G, Table 2). This observation is contrary to previous studies showing that *Slc30a1* expression is induced by the metal transcription factor 1 (MTF1) in response to Zn.^53^ However, as mice were chronically fed different TE diets it is difficult to interpret which adaptive mechanisms have already taken place to achieve a novel balance.

Surprisingly, in female mice cerebellar Mn concentrations were significantly influenced by dietary intervention even though Mn was not modulated in the three groups. In comparison to adequately supplied animals, Mn concentrations in both −TE and +TE_aa_ groups were elevated in female mice independent of their age (Fig. 2A, Table 2). Possibly, this elevation results from shared transporters of Mn with Fe and/or Zn. Total serum Zn concentrations were neither influenced by sex nor by diet but lower serum concentrations of free Zn were detected in female mice compared to males. Regarding Fe, serum transferrin levels showed a comparable pattern in female mice, with lowest concentrations in +TE groups and higher concentrations in −TE and +TE_aa_ groups.^8^ A possible candidate transporter for interactions of TEs discussed in literature is the divalent metal transporter DMT1. It has already been reported that Fe deficiency can result in increased Mn concentrations in rat brain most probably involving DMT1.^54^ However, herein *Dmt1* mRNA expression was unaffected by diet (Supplementary Table S3) but effects on protein concentrations or localization cannot be excluded. In addition, expression of *Slc39a14*, encoding ZIP14, was downregulated in adult mice of the age-adjusted groups [Fig. 3H (−TE vs. +TE_aa_)]. This divalent metal transporter is known to not only transport Fe^2+^ and Zn^2+^ but also Mn^2+^.^55^ Silencing RNA (siRNA)-mediated knockdown analysis of neuronal *Slc39a14* revealed its particular role as Mn importer as it was associated with decreased cellular Mn uptake.^56,57^ Furthermore, ZIP14 is mainly located at the basal membrane of brain endothelial cells and hence may contribute to the import of Mn into the brain.^57^ Although there is some evidence for the involvement of CP in the deposition of Mn in the brain, Mn concentrations did not correlate with *Cp* mRNA expression patterns (Supplementary Fig. S2).^58^ Based on this, it can be hypothesized that Fe deficiency might be the driving factor for higher Mn concentrations in the cerebellum of −TE mice while downregulation of ZIP14 might explain higher Mn concentrations in +TE_aa_ groups. Overall, changes in the dietary TE supply caused only mild effects on cerebellar TE concentrations, which emphasizes the tight regulation of TE homeostasis in the brain.

### Dietary TE supply did not affect markers of genomic stability in the cerebellum

**Fig. 4:**
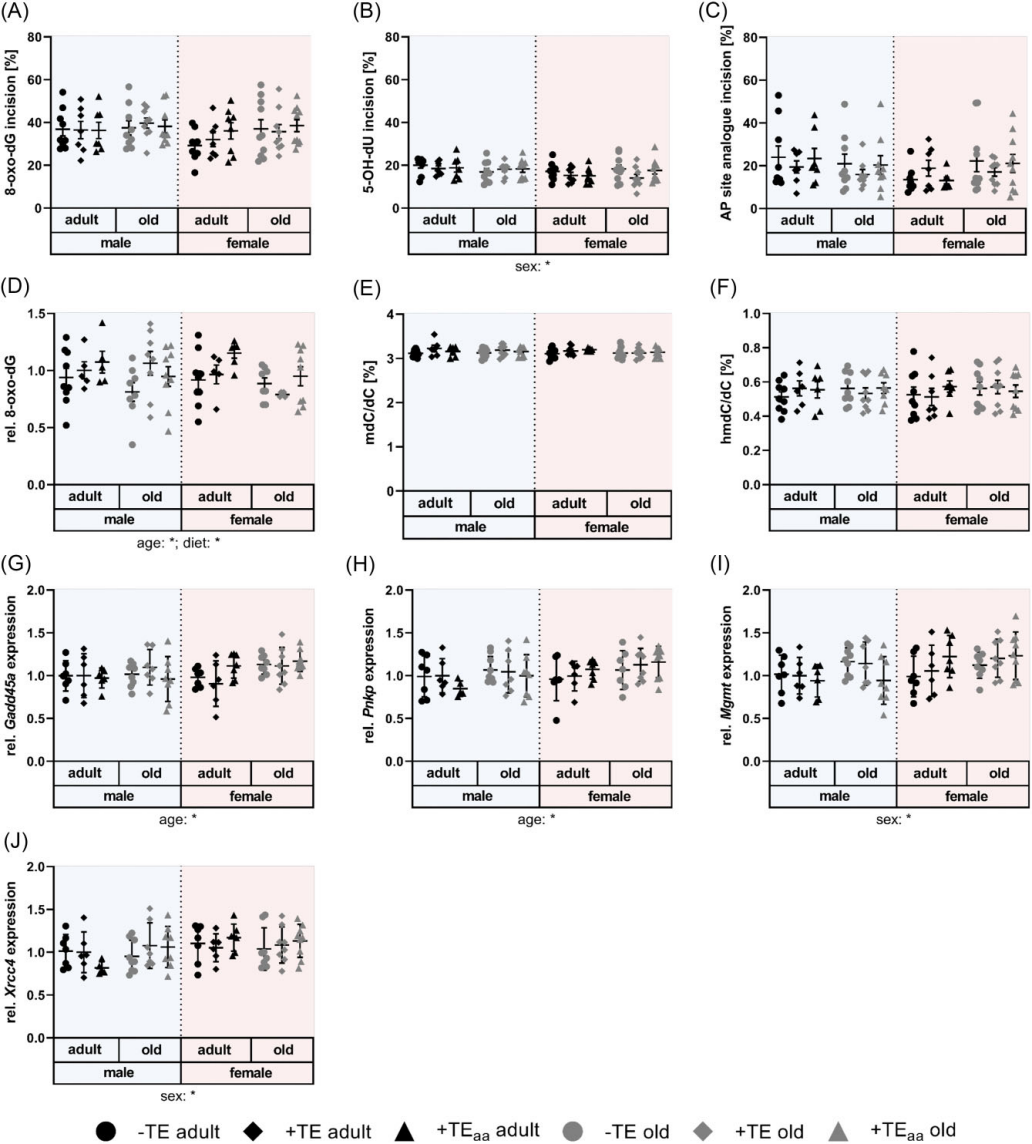
Genomic stability and epigenetic markers in murine cerebellum. Selected markers for genomic stability and global DNA (hydroxy)methylation analysed in cerebellum of adult (30 wk) and old (66 wk) male and female C57BL/6Jrj mice receiving −TE, +TE, or +TE_aa_ diet for 26 wk. BER incision activity determined toward an 8-oxo-dG- (A), a 5-OH-dU- (B), and an AP site analogue- (C) containing oligonucleotide by non-radioactive incision activity assay. Relative 8-oxo-dG levels (D) analysed by ELISA while DNA (hydroxy)methylation was quantified via HPLC–MS/MS (E, F). Gene expression levels of *Gadd45a* (G), *Pnkp* (H), *Mgmt* (I), and *Xrcc4* (J) measured via RT-qPCR and normalized to composite factor based on reference genes *Gapdh* and *Rpl13a* with variances expressed as fold changes compared to +TE male adults (mean +TE male adult = 1). Data points shown with mean ± SEM or SD (gene expression data). Statistical testing based on three-way ANOVA and Bonferroni *post hoc* test with *P < 0.05. Detailed results of statistical testing summarized in Supplementary Table S3.

To compare susceptibility of different organs to incur genomic instability, endpoints related to genomic integrity were measured in murine cerebellum and compared to respective results from liver tissue. While incision activities for 8-oxo-dG, 5-OH-dU and AP sites in hepatic tissue from the same animal experiment were increased in female mice, incision activities in the cerebellum were not affected by sex, diet, or age of the animals (Fig. 4A–C).^8^ Additionally, expression levels of a set of genes involved in BER, the main repair pathway for the investigated lesions, were not affected by the examined parameters age, diet and sex (Supplementary Table S3). In general, a reduced repair capacity for DNA damage with age is hypothesized. As was shown by other groups, ageing can have different impacts on repair protein activity across various brain tissue types.^59,60^ There are only few studies examining activities of BER proteins in distinct brain regions but Imam *et al*. described an increase of nuclear and mitochondrial incision activity for 8-oxo-dG in the cerebellum with age.^59^ One possible reason, for the inconsistence with our results could be that the aged animals were 12 wk older compared to the present experiment. Furthermore, for the present method protein extracts are prepared from native cerebellar tissue so that no difference between incision activity in nucleus and mitochondria can be measured. In the present study, no age-related increase in oxidatively damaged guanine was observed, nor did dietary TE modulation cause deleterious effects on 8-oxo-dG levels (Fig. 4D). However, in the literature, higher 8-oxo-dG concentrations have been extensively described in old mice and rats.^61–64^ In contrast, herein a trend for lower levels of 8-oxo-dG in old mice was detected. At 30 wk of age mice are regarded at the peak of mature adulthood and still reproductively active and therefore represent human adults aged around 30 yr. Mice aged 66 wk, however, can be considered at the upper age limit for middle aged rather than truly old mice. Here, first biomarkers indicate senescent changes. This group corresponds to humans aged around 50 yr old.^65^ Set in context with existing literature apparently, the biomarker of cerebellar 8-oxo-dG only changes at a later life stage in mice so that no changes were detected in this study.

Furthermore, neither age, diet, nor sex showed effects on global DNA methylation and hydroxymethylation status (Fig. 4E, F). This is in contrast to the liver, where an increase in global DNA hydroxymethylation was detected in old mice.^8,66^ Again, changes of DNA methylation might take place in time spans larger than the age differences covered in this study. Investigations of younger mice measured significant differences in hmdC in cerebella of mice 7 d and 6 wk old with a further increase in 1-yr-old mice.^67^ A second study by Lardenoije *et al.* showed a significant increase of hmdC between mice 1 and 2 yr old.^68^ Out of the 52 genes analysed involved in DNA damage response and DNA repair, only four showed significant changes, two of them were age- [growth arrest and DNA damage-inducible 45 alpha (*Gadd45a*) and polynucleotide kinase 3ʹ-phosphatase(*Pnkp*)], and the other two sex-dependent (O-6-methylguanine-DNA methyltransferase (*Mgmt*) and X-ray repair complementing defective repair in Chinese hamster cells 4 (*Xrcc4*)] (Fig. 4G–J). However, effects were too small to be considered as of physiological relevance, which is supported by the lack of substantial effects on markers of genomic stability.

## Concluding remarks

Overall, the implemented modifications in dietary TE supply did not cause substantial TE dyshomeostasis in the cerebellum. However, in old mice concentrations of Cu and Fe were moderately but significantly increased. Regarding sex-specific effects Se concentrations were higher in female animals. Accordingly, gene expression levels of the investigated TE-related transport proteins were also more strongly affected by age and sex than by dietary TE supply. In the cerebellum neither diet, sex, nor age affected genomic stability-related parameters within the time span studied. To conclude, both slightly reduced supply of TEs as well as age-adapted increase of dietary Se und Zn did not considerably impact cerebellar TE concentrations. This underlines the ability of the cerebellum to counteract disturbed nutritional availability of certain TEs to ensure cerebellar TE homeostasis.

## Supplementary Material

mfae003_Supplemental_Files

## Data Availability

All data are available on request.
