# Supplementary material for: Long-term suboptimal dietary trace element supply does not affect trace element homeostasis in murine cerebellum
Source: Metallomics. 2024 Jan 31;16(2):mfae003. doi: 10.1093/mtomcs/mfae003 (PMC10873500; doi:10.1093/mtomcs/mfae003)
Supplement: mfae003_Supplemental_Files [file mfae003_supplemental_files.zip › suppl_data Supplementary Information.docx]

**Supplementary Table S1: Group size specification.** Exact number of animals and samples for each endpoint with *n* after outlier identification stated in brackets. Samples of all animals were subjected to element analyses *via* ICP-MS/MS whereas due to tissue volume limitations some endpoints could not be performed on all animals.

| **Endpoint** | **Male** | | | | | | **Female** | | | | | |
| --- | --- | --- | --- | --- | --- | --- | --- | --- | --- | --- | --- | --- |
|  | **Adult** | | | **Old** | | | **Adult** | | | **Old** | | |
|  | **-TE** | **+TE** | **+TE_aa_** | **-TE** | **+TE** | **+TE_aa_** | **-TE** | **+TE** | **+TE_aa_** | **-TE** | **+TE** | **+TE_aa_** |
| feeding regime | 9 | 8 | 8 | 9 | 9 | 9 | 9 | 8 | 8 | 10 | 9 | 10 |
| TE analysis |  |  |  |  |  |  |  |  |  |  |  |  |
| Cu | 9 | 8 | 8 | 9 | 9 | 9 (8) | 9 | 8 (7) | 8 | 10 (9) | 9 (8) | 10 (9) |
| Fe | 9 | 8 | 8 | 9 | 9 | 9 | 9 | 8 (7) | 8 (7) | 10 | 9 (8) | 10 |
| Mn | 9 | 8 | 8 | 9 | 9 | 9 | 9 | 8 (6) | 8 (7) | 10 (9) | 9 (8) | 10 |
| Se | 9 | 8 | 8 | 9 | 9 | 9 (8) | 9 | 8 (7) | 8 | 10 (9) | 9 | 10 |
| Zn | 9 | 8 | 8 | 9 (8) | 9 | 9 | 9 | 8 (7) | 8 | 10 (9) | 9 | 10 |
| RT-qPCR | 7 | 6 | 6 | 9 | 7 | 8 | 7 | 6 | 7 | 8 | 7 | 9 |
| BER incision activity analysis |  |  |  |  |  |  |  |  |  |  |  |  |
| 5-OH-dU incision | 9 | 7 | 7 | 9 | 9 | 9 | 9 | 7 | 8 | 10 | 9 | 10 |
| 8-oxo-dG incision | 9 | 7 | 7 | 9 | 9 | 9 | 9 | 7 | 8 | 10 | 9 | 10 |
| AP site analogue incision | 9 | 7 | 7 | 9 | 9 (8) | 9 | 9 (8) | 7 | 8 (7) | 10 | 9 | 10 |
| oxidative DNA damage analysis | 9 | 5 | 5 | 8 | 8 | 9 | 9 | 5 | 6 | 8 | 7 (6) | 9 |
| global (hydroxy-)methylation analysis |  |  |  |  |  |  |  |  |  |  |  |  |
| hydroxymethylation | 9 | 6 | 6 | 9 | 9 | 8 | 9 | 7 | 6 | 9 | 9 | 9 |
| methylation | 9 | 6 | 6 | 9 | 9 | 8 | 9 | 7 | 6 (5) | 9 | 9 | 9 |

**Supplementary Information: RNA Isolation, Reverse Transcription, and qPCR.**

Cerebellar RNA was isolated from snap-frozen tissue and stored at -80 °C for up to three months. RNA was isolated using TRIzol Reagent (Thermo Fisher Scientific) according to the manufacturer’s instructions. For sample lysis, 1 mL TRIzol Reagent was added to 15-25 mg frozen cerebellar tissue, homogenized by bead beating using zirconium beads and incubated for 5 min. Afterwards, 0.2 mL chloroform were added and samples were thoroughly mixed, further incubated for 3 min on ice and centrifuged for 15 min at 12 000 x *g* at 4 °C for phasing. The aqueous phase containing the RNA was transferred into a new tube, 0.5 mL isopropanol were added and incubated for 10 min at 4 °C prior to centrifugation at 12 000 x *g* at 4 °C for 10 min for RNA precipitation. The RNA pellet was resuspended in 1 mL 75 % ethanol following centrifugation at 7500 x *g* at 4 °C for 5 min and air-dried. RNA was resuspended in RNase-free water and incubated at 55 °C for 5-10 min. Quantification of RNA yield was determined using NanoDrop Spectrophometer (Thermo Fisher Scientific) (Supplementary Table S2). All samples used for further analysis showed an absorbance ratio of A260/280 between 1.93 and 2.05 whereas A260/230 was in between 0.67 and 2.42.

For DNA digestion 1 U DNase I (Thermo Fisher Scientific) was incubated with 4 µg of RNA at 37 °C for 30 min. To stop the reaction, 1 µL of 50 mM EDTA was added and temperature increased to 65 °C for 10 min. Those 4 µg of DNA-free RNA were incubated with cDNA SuperMix (qScript cDNA synthesis, Quanta BioSciences) in a final volume of 20 µL. For reverse transcription, the following temperature program was used: 5 min at 25 °C, 30 min at 42 °C, 5 min at 85 °C, cooled to 4 °C. 50 ng cDNA for each gene of interest were amplified using 1x PerfeCTa SYBR Green Supermix (Quanta Biosciences) and 250 nM priming oligonucleotides in a total volume of 10 µL. As DNA digestion and reverse transcription, also qPCR was conducted in a CFX Connect Real-time PCR Detection System (Bio-Rad Laboratories) using the following cycling program: 3 min at 95 °C, followed by 41 cycles of 15 s at 95 °C, 20 s at either 60 °C or 55 °C (Supplementary Table S3), and 30 s at 72 °C. Primers were tested for specificity *in silico* using Primer-BLAST. Furthermore, amplicon lengths were verified via gel electrophoresis (2 % agarose gel, stained with GelRed) and the respective melting temperature was checked with the used CFX Connect Real-time PCR Detection System. Furthermore, primer efficiencies were assessed by analysing a standard curve of 10x diluted PCR products and calculated from the slope of the standard curve. Only primers with a calculated efficiency of 80-120 % were used for RT-qPCR analysis. More detailed information on parameters of qPCR validation and data analysis is available on request. As potential reference genes *Actb*, *Gapdh* and *Rpl13a* were tested. However, due to large fluctuations, *Actb* was dismissed and only the more stable *Gapdh* and *Rpl13a* were used as reference genes. A normalization factor for each sample was calculated based on the geometric mean of the two reference genes. From the Cq values, copy numbers were calculated using DNA standards from cDNA amplified by PCR for each gene of interest. Due to limited amounts of sample material, no technical replicates were measured. Finally, relative gene expression was obtained by relating each normalized copy number to the mean of all male adult animals receiving adequate amounts of TEs.

**Supplementary Table S2. RT-qPCR target and oligonucleotide information.**

| **Gene, encoded protein** | **RefSeq-ID** | **Sequence 5’ 🡪 3’** | **Melting temperature** | **Amplicon length [bp]** | **Location** | **Additional splice variants** |
| --- | --- | --- | --- | --- | --- | --- |
| **Apex1**, apurinic/apyrimidinic endonuclease 1 | NM_009687.2 | CCCTCCAGATCAGAAAACCTC  AGGCAGCTCTTGCAGTTCAG | 55 °C | 196 | Exon 3-4 | 4 |
| **Aplf**, aprataxin and PNKP like factor | NM_024251.4 | GACAGGCCCGAGTGTCCCTA  CTGGGTTGCCCGACATCATCG | 60 °C | 131 | Exon 8-10 | 3 |
| **Aptx**, aprataxin | NM_025545.4 | CTGCGCTTCCGATTGGGCTA  GTCACTCTGCCGGCTTCCTG | 60 °C | 182 | Exon 6-8 | 3 |
| **Atm**, ataxia telangiectasia mutated | NM_007499.3 | CAGCTTGTGAAGGGCCGTGA  CGAGAACACCGCTTCGCTGA | 60 °C | 163 | Exon 56-58 | 2 |
| **Atp7a**, ATPase Copper transporting alpha polypeptide | NM_001109757.2 | GTCTCTGGGATGACCTGTGCT  TCTTACTTCTGCCTTGCCAGCC | 60 °C | 114 | Exon 5-6 | 1 |
| **Atp7b**, ATPase Copper transporting beta polypeptide | NM_007511.2 | CAGATGTCAAAGGCTCCCATTCAG  CCAATGACGATCCACACCACC | 60 °C | 110 | Exon 11/12-12 | 1 |
| **Atr**, ataxia telangiectasia and Rad3 related | NM_019864.1 | CCGTCTCTGGAGCGGCATAC  GGCAAGGAGCACTTGGGAGT | 60 °C | 159 | Exon 13-14 | 2 |
| **Atrip**, ATR interacting protein | NM_172774.3 | GTGCTAACACGCCTCTCTTCCA  CTGCATCCAGCCATCAGCCA | 60 °C | 155 | Exon 5-6 | 3 |
| **Bak1**, BCL2-antagonist/killer 1 | NM_007523.3 | GCCCTGTACGTCTACCAGCGT  CGGTCAGGATGGGGTCTCTACG | 60 °C | 176 | Exon 4-5 | - |
| **Bax**, BCL2-associated X protein | NM_007527.3 | CGGGTGGCAGCTGACATGTTT  CCAGCCACCCTGGTCTTGGAT | 60 °C | 195 | Exon 4-5 | 3 |
| **Bcl2**, B cell leukemia/lymphoma 2 | NM_009741.5 | GGTGGGGTCATGTGTGTGGAG  CGCATGCTGGGGCCATATAGTT | 60 °C | 161 | Exon 1-2 | 2 |
| **Brca1**, breast cancer 1, early onset | NM_009764.3 | CCGGAACCGTGTCAGAAGGC  CGAGGTTGGGTCTGCCTGTTT | 60 °C | 129 | Exon 6-7 | 2 |
| **Brca2**, breast cancer 2, early onset | NM_009765.3 | GAGCCGGTTACCTGTGGACC  CAGAAACGTCGTGAGCCGGT | 60 °C | 181 | Exon 13-14 | 2 |
| **Cdkn1a**, cyclin-dependent kinase inhibitor 1A (P21) | NM_007669.5 | GACAGTGAGCAGTTGCGCC  CTCAGACACCAGAGTGCAAGAC | 55 °C | 288 | Exon 2 | 4 |
| **Chek1**, checkpoint kinase 1 | NM_007691.5 | GGATCACCATCCCAGACATTAAG  AGACTCTGACATACCACCTGATGT | 55 °C | 101 | Exon 7-8 | 3 |
| **Chek2**, checkpoint kinase 2 | NM_016681.4 | GAACTCGGTGACCCTCCCTG  GACAGGCGTTTTGTGCTCGG | 60 °C | 99 | Exon 14-15 | 1 |
| **Cp**, Ceruloplasmin | NM_001276248.1 | GTACTACTCTGGCGTTGACCC  TTGTCTACATCTTTCTGTCTCCCA | 55 °C | 114 | Exon 9-9/10 | 9 |
| **Ctcf**, CCCTC-binding factor | NM_181322.3 | CTGCAGCCACTGCGACAAGA  GCCATCTGGACCAGCACAGTT | 60 °C | 175 | Exon 9-10 | 1 |
| **Ctr1**, solute carrier family 31, member 1 | NM_175090.4 | ACCATGCCACCTCACCACCA  GCTCCAGCCATTTCTCCAGGT | 60 °C | 161 | Exon 2-3/4 | - |
| **Dmt1**, solute carrier family 11 (proton-coupled divalent metal ion transporters), member 2 | NM_001146161.1 | CTCAGCCATCGCCATCAATCTC  TTCCGCAAGCCATATTTGTCCA | 55 °C | 117 | Exon 6-7/8 | 2 |
|  |  |  |  |  |  |  |
| **Dnmt1**, DNA methyltransferase (cytosine-5) 1 | NM_001199432.1 | GGAGTCACACACCGTTCCCG  CACACTCGGGGCACTTTGGT | 60 °C | 101 | Exon 13-15 | 4 |
| **Ercc1**, excision repair cross-complementing rodent repair deficiency, complementation group 1 | NM_007948.2 | GAACTTCGCCCTTCGTGTGC  CAGGGTGCAGTCAGCCAAGA | 60 °C | 103 | Exon 5-6 | 3 |
| **Ercc4**, excision repair cross-complementing rodent repair deficiency, complementation group 4 | NM_015769.2 | CCCGAAGAACGGGAAGGCAG  GGGAGCTCGCTCCGAAACTC | 60 °C | 161 | Exon 10-11 | - |
| **Ercc6**, excision repair cross-complementing rodent repair deficiency, complementation group 6 | NM_001081221.2 | GGGACCACCACCATAGCGTC  GTTGGCACCGGTCAGGTTCA | 60 °C | 123 | Exon 14-15 | 1 |
| **Ercc8**, excision repair cross-complementing rodent repair deficiency, complementation group 8 | NM_028042.4 | CTTGCGTAGAGCGCAGTCCA  GGCTGTCTGCTGGCGTTCTC | 60 °C | 189 | Exon 1-3 | 2 |
| **Fas**, Fas (TNF receptor superfamily member 6) | NM_007987.2 | CTGTCCTGCCTCTGGTGCTTG  CCGCCTCCTCAGCTTTAAACTCTC | 60 °C | 92 | Exon 1-2 | 4 |
| **Fen1**, flap structure specific endonuclease 1 | NM_007999.6 | GGTGGAGGAGAGGTGACTAG  GCACAACTACTGGACTCAGCAG | 55 °C | 212 | Exon 3 | 4 |
| **Fth1**, ferritin heavy polypeptide 1 | NM_010239.2 | CGCCAGAACTACCACCAGGA  TTCTTCAGAGCCACATCATCTCGG | 60 °C | 125 | Exon 1-2 | - |
| **Gadd45a**, growth arrest and DNA-damage-inducible 45 alpha | NM_007836.1 | AGAAGACCGAAAGGATGGACAC  CACGGATGAGGGTGAAATGGAT | 55 °C | 207 | Exon 1-2 | 2 |
| **Gadd45g**, growth arrest and DNA-damage-inducible 45 gamma | NM_011817.2 | CGGGAAAGCACTGCACGAAC  CACATTGTCAGGGTCCACATTCAG | 60 °C | 106 | Exon 2-3 | 1 |
| **Gapdh**, glyceraldehyde-3-phosphate dehydrogenase | NM_008084.3 | GGGTGTGAACCACGAGAAAT  GTCTTCTGGGTGGCAGTGAT | 60 °C | 162 | Exon 4-5 | 4 |
| **Hamp**, hepcidin antimicrobial peptide | NM_032541.2 | GCCTGAGCAGCACCACCTAT  TGCAACAGATACCACACTGGGA | 60 °C | 189 | Exon 1-3 | - |
| **Heph**, hephaestin | NM_010417.2 | CTCTTGCTCCATGGACCCAC  ATCTGGGCCACTTCCTGGCT | 55 °C | 151 | Exon 6-6/7 | 3 |
| **Lat1**, solute carrier family 7 (cationic amino acid transporter), member 5 | NM_011404.3 | TAAAGGCTGCGACCCGTGTG  ACGCATCACCTTGTCCCATGTC | 60 °C | 117 | Exon 2-2/3 | - |
| **Lat2**, solute carrier family 7 (cationic amino acid transporter), member 8 | NM_016972.2 | CCTGCTCTTCACATGCCTCTC  ATCTGTCCTGCAACCGTTACCC | 55 °C | 120 | Exon 8/9-9 | - |
| **Lig1**, ligase I, DNA, ATP-dependent | NM_010715.3 | CACCCCCAAAGTCCAGAAGCC  GGGGGAGCTGCAGTTGAAGAC | 60 °C | 103 | Exon 4-5 | 5 |
| **Lig3**, ligase III, DNA, ATP-dependent | NM_001291246.1 | GCAGAGGTGGCATCAAACCGA  GTGGCAAGTAGAGCCGCACC | 60 °C | 138 | Exon 3-4 | 5 |
| **Lig4**, ligase IV, DNA, ATP-dependent | NM_176953.4 | GGGACTGATTTCAGGTGGCA  GCGACAACAAATCCTCCGGT | 60 °C | 183 | Exon 1-2 | 1 |
| **Lrp1**, low density lipoprotein receptor-related protein 1 | NM_008512.2 | CCAACCAGGCTACAAGACCTC  AATCCATGCAGTCGGTGTCCC | 55 °C | 117 | Exon 20/21-21 | - |
| **Lrp2**, low density lipoprotein receptor-related protein 2, megalin | NM_001081088.2 | CGCCAGTCAGTGGCCAAGAA  AAAGCCTGAACCGCAGGACC | 60 °C | 152 | Exon 1/2-3 | - |
| **Lrp8**, low density lipoprotein receptor-related protein 8, apolipoprotein E receptor 2 (ApoER2) | NM_001080926.1 | GGACAGACTCAGGCAATAAGACCA  GACCAGTACATGAACCCTCGCAG | 60 °C | 132 | Exon 10-10/11 | 5 |
| **Mct8**, solute carrier family 16 (monocarboxylic acid transporters), member 2 | NM_009197.2 | CTTCACCAGCTCCCTAAGCCT  AGTAGTGGCCCAGGATGACGA | 60 °C | 104 | Exon 2/3-3 | - |
|  |  |  |  |  |  |  |
| **Mdm2**, transformed mouse 3T3 cell double minute 2 | NM_010786.4 | CAGAGACGCCCTCGCATCAG  GAGAGCTCGTGCCCTTCGTC | 60 °C | 160 | Exon 9-10 | 2 |
| **Nis**, solute carrier family 5 (sodium iodide symporter), member 5 | NM_053248.2 | CTAGAACTGCGCTTCAGCCGA  ACCCGGTCACTTGGTTCAGGA | 60 °C | 124 | Exon 2-3/4 | - |
| **Nthl1**, nth (endonuclease III)-like 1 (E. coli) | NM_008743.2 | GATCGCGTCGGAAGGGTGTAG  GTTCTGGGGCTCCCAAACGG | 60 °C | 187 | Exon 1-2 | 1 |
| **Oatp1c1**, solute carrier organic anion transporter family member 1c1 | NM_021471.3 | GCACCGTTCAGTTCAATTCTCTA  GAATGTTGATGAGCCCAATGAC | 55 °C | 117 | Exon 8-8/9 | 1 |
| **Ogg1**, 8-oxoguanine DNA-glycosylase 1 | NM_010957.4 | GTGACTACGGCTGGCATCC  AGGCTTGGTTGGCGAAGG | 60 °C | 154 | Exon 5-7 | 1 |
| **Palb2**, partner and localizer of BRCA2 | NM_001081238.2 | GCTGAAGTCCAAGGGACGCA  GCTCTCTTTGGCACAAGGTTGAC | 60 °C | 198 | Exon 10-12 | 4 |
| **Parg**, poly (ADP-ribose) glycohydrolase | NM_011960.3 | CACACGACCCCAGAACTTGA  GTTGGGCCTCTGCTTCTTCA | 60 °C | 122 | Exon 6-8 | 4 |
| **Parp1**, poly (ADP-ribose) polymerase family, member 1 | NM_007415.3 | GGCAGCCTGATGTTGAGGTG  GCGTACTCCGCTAAAAAGTCAC | 55 °C | 160 | Exon 2-3 | 2 |
| **Pnkp**, polynucleotide kinase 3’-phosphatase | NM_001290764.1 | CCAGATGTCCCAAGTCGGGC  GTTGTGGCGTGCCTGCTCTA | 60 °C | 105 | Exon 13-14 | 8 |
| **Polb**, polymerase (DNA directed), beta | NM_011130.2 | GCGCAGAGTCGAGTGGAGAC  CCTTCTCGCTGGGAAGCTGG | 60 °C | 186 | Exon 10-12 | 2 |
| **Rad50**, RAD50 double strand break repair protein | NM_009012.2 | GGCGTGCGAAGTTTTGGGATAG  GCCCGCACGTCTGTTTCTTGA | 55 °C | 209 | Exon 1-3 | 3 |
| **Rad51**, RAD51 recombinase | NM_011234.5 | CACGGTTAGAGCAGTGTGGCA  AGTTGCCGTGGTGAAACCCA | 60 °C | 194 | Exon 2-4 | 3 |
| **Rpl13a**, ribosomal protein L13A | NM_009438.5 | GTTCGGCTGAAGCCTACCAG  TTCCGTAACCTCAAGATCTGCT | 60 °C | 158 | Exon 5-6/7 | 7 |
| **Selenbp1**, selenium binding protein 1 | NM_009150.3 | CAGGTCATCCACAGGTTGCCCA  ACGTAGATGCGGGAGGAGATGAG | 60 °C | 146 | Exon 3/4-4 | - |
| **Sepp1**, selenoprotein P | NM_001042613.1 | CTCATCTATGACAGATGTGGCCGT  AAGACTCGTGAGATTGCAGTTTCC | 60 °C | 138 | Exon 3/4-4/5 | 2 |
| **Sirt6**, sirtuin 6 | NM_181586.4 | ACGCGGATAAGGGCAAGTG  CTCCCACACCTTGCGTTC | 55 °C | 74 | Exon 1-2 | 3 |
| **Slc30a1**, solute carrier family 30 (zinc transporter), member 1 (ZnT1) | NM_009579.3 | CACGACTTACCCATTGCTCAAG  CTTTCACCAAGTGTTTGATATCGATT | 55 °C | 86 | Exon 2 | - |
| **Slc30a10**, solute carrier family 30, member 10 (ZnT10) | NM_001033286.2 | ACTGGCAGTGCTACATTGACCC  CAGCTGGCTCATCAGCTCTTC | 60 °C | 149 | Exon 3-3/4 | - |
| **Slc30a3**, solute carrier family 30 (zinc transporter), member 3 (ZnT3) | NM_011773.3 | TGTGCCAATCTGCTAATGGCCT  CCGGACACTCGTGTTTCCCA | 60 °C | 135 | Exon 4/5-5 | 1 |
| **Slc30a4**, solute carrier family 30 (zinc transporter), member 4 (ZnT4) | NM_011774.3 | GCTGAAGCAGAGGAAGGTGAA  TCTCCGATCATGAAAAGCAAGTAG | 55 °C | 72 | Exon 1 | 1 |
| **Slc30a5**, solute carrier family 30 (zinc transporter), member 5 (ZnT5) | NM_022885.2 | CCACTAAGGACCTTGCTGCTGT  TGTCCTTCCGGGTGTTCAGC | 60 °C | 194 | Exon 4/5-6/7 | - |
| **Slc30a6**, solute carrier family 30 (zinc transporter), member 6 (ZnT6) | NM_001252478.1 | GCCCCGCATGAATCCGTTTG  ACGTCATCAGGGCAATGGCA | 60 °C | 134 | Exon 10-11 | 1 |
| **Slc30a9**, solute carrier family 30 (zinc transporter), member 9 (ZnT9) | NM_178651.4 | GGAAGCCTTGAAGCTCTTGCCA  GGAACGTGGCTTTGTGTTTCCC | 60 °C | 135 | Exon 6-7/8 | 1 |
| **Slc39a1**, solute carrier family 39 (zinc transporter), member 1 (Zip1) | NM_013901 | TCCTCAAGGTCATTCTGCTCCTA  CCCTTTCTCTTGAAGCACCTTAGA | 55 °C | 87 | Exon 4 | 1 |
| **Slc39a10**, solute carrier family 39 (zinc transporter), member 10 (Zip10) | NM_001356416.1 | TGGCTTACATAGGAATGCTCATAGG  TGCGAAGATCCAGAGTGTGATG | 55 °C | 74 | Exon 8 | 2 |
| **Slc39a11**, solute carrier family 39 (metal ion transporter), member 11 (Zip11) | NM_001166503.1 | GCTGCTGGTGCCATGGTCTA  CGGAGTCCCACGGTTTGTCA | 60 °C | 161 | Exon 8-9 | 9 |
| **Slc39a12**, solute carrier family 39 (zinc transporter), member 12 (Zip12) | NM_001012305 | AACCATCCAACTGAAAGGCCCA  GTGCAGGCTATCTCCAACCAG | 55 °C | 133 | Exon 8/9-10 | 8 |
| **Slc39a13**, solute carrier family 39 (metal ion transporter), member 13 (Zip13) | NM_001290765.2 | CCCATGAGGTGGGTGACTTTGC  GTACAGATGGCAAAGCAGGCCC | 60 °C | 121 | Exon 6/7-7 | 1 |
| **Slc39a14**, solute carrier family 39 (zinc transporter), member 14 (Zip14) | NM_144808.4 | GAGCCAACTGATAATCCATTGCT  GTCAACGGCCACATTTTCAA | 55 °C | 68 | Exon 8 | 2 |
| **Slc39a2**, solute carrier family 39 (zinc transporter), member 2 (Zip2) | NM_001039676.2 | GTGGCTATAGGCCATTCCCG  GGCTGACAACAGGCACTGAG | 55 °C | 195 | Exon 4 | - |
| **Slc39a3**, solute carrier family 39 (zinc transporter), member 3 (Zip3) | NM_134135.3 | CCGGTGGCACCTTCCTGTTTG  AGGGACGTGCTCTGTGTCCTT | 60 °C | 175 | Exon 2 | 1 |
| **Slc39a4**, solute carrier family 39 (zinc transporter), member 4 (Zip4) | NM_028064 | CTCTGCAGCTGGCACCAA  CACCAAGTCTGAACGAGAGCTTT | 55 °C | 71 | Exon 8-8/9 | - |
| **Slc39a5**, solute carrier family 39 (metal ion transporter), member 5 (Zip5) | NM_001136237.1 | CAGTGGCCTCAGCACTACCC  CCAGGGCTCCAGAAACGAGG | 60 °C | 134 | Exon 8-9 | 2 |
| **Slc39a6**, solute carrier family 39 (metal ion transporter), member 6 (Zip6) | NM_139143 | GCCACAGCCAGCGCTA  ATCACCATCCAGGCCAATGT | 55 °C | 70 | Exon 6-6/7 | - |
| **Slc39a7**, solute carrier family 39 (zinc transporter), member 7 (Zip7) | NM_008202.2 | CGGTCTGGCCATTGGTGCTT  TCACGAGTTGCAGACGCATCG | 60 °C | 161 | Exon 6-7 | 1 |
| **Slc39a8**, solute carrier family 39 (metal ion transporter), member 8 (Zip8) | NM_001135149.1 | CTAACGGACACATCCACTTCGA  CCTTCAGACAGGTACATGAGCTT | 55 °C | 87 | Exon 5-5/6 | 2 |
| **Slc39a9**, solute carrier family 39 (zinc transporter), member 9 (Zip9) | NM_026244 | ATGCTACACAAGGCTCCAGCAG  GGTGCTGCCAATGCAAAGACC | 60 °C | 110 | Exon 5-5/6 | - |
| **Slc40a1**, solute carrier family 40 (iron-regulated transporter), member 1, ferroportin (Fpn) | NM_016917.2 | CTGGTGGTTCAGAATGTGTCCGT  AGCAGACAGTAAGGACCCATCCA | 60 °C | 112 | Exon 4-4/5 | - |
| **Slc48a1**, solute carrier family 48 (heme transporter), member 1 | NM_026353.4 | ATTGGCCATCACCCAGCATCAG  CTGATGTCCGCAAAGTCAGCC | 60 °C | 141 | Exon 2-3 | - |
| **Tdg**, thymine DNA glycosylase | NM_172552.4 | GGGCAGCAAGGATCTGTCTAG  CACCGCTATTCGTGGCTGAT | 60 °C | 91 | Exon 5-6 | - |
| **Tdp1**, tyrosyl-DNA phosphodiesterase 1 | NM_028354.5 | CTCCGGGCAAGAGTGCAGTT  GGCCGGATGTCTCTGCTGAC | 60 °C | 174 | Exon 10-13 | 2 |
| **Tet1**, tet methylcytosine dioxygenase 1 | NM_001253857.2 | TGCTGGAGACTGTCGACTTG  GGACGTGGAGTTGTTCATCC | 55 °C | 188 | Exon 11-12 | 5 |
| **Tet2**, tet methylcytosine dioxygenase 2 | NM_001040400.2 | ATCATGTTGTGGGACGGAATCC  CATGCTCCAAGAACAACCAAAAGA | 55 °C | 192 | Exon 6-7 | 2 |
| **Trf**, transferrin | NM_133977.2 | CCGGCTTGCCTGTGTGAAGA  ACGGGCTTCAGGTTGTTCGG | 60 °C | 135 | Exon 2-3 | - |
|  |  |  |  |  |  |  |
| **Trfc**, transferrin receptor | NM_011638.4 | GGCTGAAACGGAGGAGACAGA  CTGGCTCAGCTGCTTGATGGT | 60 °C | 151 | Exon 3-3/4 | 1 |
| **Trp53**, transformation related protein 53 | NM_011640.3 | CACAGTCGGATATCAGCCTCG  CATGCAGTGAGGTGATGGCA | 60 °C | 104 | Exon 2-4 | 4 |
| **Trpml1**, mucolipin 1 (Mcoln1) | NM_053177.1 | GGATGGCAGCGCCAGTTACA  TTCGCCCACTGTGCGCTTTA | 60 °C | 176 | Exon 5-7 | - |
| **Xpa**, xeroderma pigmentosum, complementation group A | NM_011728.2 | CCACTTTGATCTGCCAACGTGTG  CCACTGTGAATGGCGTGGGT | 60 °C | 172 | Exon 3-4 | - |
| **Xrcc1**, X-ray repair complementing defective repair in Chinese hamster cells 1 | NM_009532.5 | CAGAGGCTGACCTGCCAATCC  GTGATGACGAACTGGACCCGC | 60 °C | 169 | Exon 16-18 | 2 |
| **Xrcc4**, X-ray repair complementing defective repair in Chinese hamster cells 4 | NM_028012.4 | GGAGTCTCGGCATTTCTCCC  CCAGTCTCTCAGGAGCCTTTC | 60 °C | 172 | Exon 2-3 | 2 |

**Supplementary Table S3. Overview of mean values and statistical testing of determined parameters in murine cerebellum.** Values shown as mean ± standard error of the mean or standard deviation (for rel. mRNA expression) and p-values for statistical analysis of adult (29 weeks) and old (66 weeks) male and female C57BL/6Jrj mice receiving -TE, +TE, or +TE_aa_ diet for 26 weeks. Statistical testing based on Three-Way ANOVA and Bonferroni post-hoc test.

| **Parameter** | **Unit** | **Male** | | | | | | **Female** | | | | | | **Statistics** | | | | | | |
| --- | --- | --- | --- | --- | --- | --- | --- | --- | --- | --- | --- | --- | --- | --- | --- | --- | --- | --- | --- | --- |
|  |  | **Adult** | | | **Old** | | | **Adult** | | | **Old** | | |  |  |  |  |  |  |  |
|  |  | **-TE** | **+TE** | **+TE_aa_** | **-TE** | **+TE** | **+TE_aa_** | **-TE** | **+TE** | **+TE_aa_** | **-TE** | **+TE** | **+TE_aa_** | **Diet** | **Sex** | **Age** | **Diet x sex** | **Diet x age** | **Age x sex** | **Diet x sex x age** |
| rel. cerebellar weight | [mg/g body weight] | 3.55 ± 0.68 | 3.42 ± 0.48 | 3.82 ± 0.33 | 2.91 ± 0.20 | 3.23 ± 0.64 | 3.35 ± 0.53 | 5.63 ± 0.65 | 4.89 ± 0.34 | 4.61 ± 1.17 | 4.04 ± 0.81 | 4.30 ± 0.44 | 4.26 ± 0.58 | 0.6724 | **<0.0001** | **<0.0001** | 0.1386 | **0.0164** | **0.0100** | 0.4554 |
|  |  |  |  |  |  |  |  |  |  |  |  |  |  |  |  |  |  |  |  |  |
| Cu | [mg kg^-1^] | 3.90 ± 0.24 | 5.69 ± 0.33 | 4.70 ± 0.29 | 5.56 ± 0.40 | 5.44 ± 0.28 | 4.50 ± 0.14 | 4.05 ± 0.12 | 4.48 ± 0.23 | 5.31 ± 0.48 | 5.33 ± 0.31 | 5.79 ± 0.12 | 5.35 ± 0.16 | **0.0073** | 0.5968 | **0.0002** | **0.0154** | **0.0007** | 0.1462 | 0.0522 |
| Fe | [mg kg^-1^] | 16.65 ± 0.89 | 19.23 ± 0.30 | 18.23 ± 0.85 | 19.33 ± 0.50 | 18.96 ± 0.72 | 18.64 ± 0.54 | 16.82 ± 0.48 | 17.13 ± 0.40 | 17.67 ± 0.70 | 19.01 ± 0.79 | 19.46 ± 0.39 | 19.63 ± 0.48 | 0.2130 | 0.5450 | **<0.0001** | 0.5283 | 0.2075 | 0.0997 | 0.2046 |
| Mn | [mg kg^-1^] | 0.37 ± 0.02 | 0.38 ± 0.01 | 0.37 ± 0.02 | 0.38 ± 0.01 | 0.34 ± 0.02 | 0.39 ± 0.01 | 0.47 ± 0.02 | 0.31 ± 0.01 | 0.39 ± 0.02 | 0.44 ± 0.01 | 0.33 ± 0.01 | 0.41 ± 0.02 | **<0.0001** | **0.0311** | 0.8914 | **<0.0001** | 0.2081 | 0.8394 | **0.0398** |
| Se | [mg kg^-1^] | 0.16 ± 0.01 | 0.19 ± 0.01 | 0.16 ± 0.01 | 0.17 ± 0.01 | 0.17 ± 0.01 | 0.16 ± 0.01 | 0.17 ± 0.01 | 0.17 ± 0.01 | 0.19 ± 0.01 | 0.19 ± 0.01 | 0.18 ± 0.01 | 0.20 ± 0.01 | 0.4087 | **0.0008** | 0.2232 | **0.0112** | 0.2228 | 0.1403 | 0.3748 |
| Zn | [mg kg^-1^] | 9.51 ± 0.52 | 10.70 ± 0.33 | 10.91 ± 0.56 | 11.30 ± 0.59 | 10.42 ± 0.36 | 11.49 ± 0.87 | 9.93 ± 0.22 | 10.67 ± 0.71 | 11.65 ± 1.01 | 9.74 ± 0.19 | 10.16 ± 0.31 | 10.36 ± 0.42 | **0.0403** | 0.3403 | 0.9565 | 0.8383 | 0.2202 | **0.0348** | 0.4573 |
|  |  |  |  |  |  |  |  |  |  |  |  |  |  |  |  |  |  |  |  |  |
| rel. Atp7a mRNA |  | 1.08 ± 0.20 | 1.00 ± 0.31 | 1.02 ± 0.18 | 1.03 ± 0.23 | 0.96 ± 0.06 | 0.98 ± 0.17 | 1.11 ± 0.27 | 1.02 ± 0.03 | 0.97 ± 0.26 | 1.06 ± 0.26 | 1.08 ± 0.17 | 1.21 ± 0.26 | 0.5888 | 0.1858 | 0.7110 | 0.8659 | 0.4477 | 0.1728 | 0.4564 |
| rel. Atp7b mRNA |  | 1.06 ± 0.19 | 1.00 ± 0.13 | 1.08 ± 0.14 | 1.04 ± 0.13 | 1.03 ± 0.12 | 1.02 ± 0.11 | 0.99 ± 0.05 | 1.07 ± 0.11 | 0.93 ± 0.16 | 1.03 ± 0.11 | 1.07 ± 0.11 | 1.04 ± 0.08 | 0.7256 | 0.5783 | 0.4844 | 0.1897 | 0.9982 | 0.2222 | 0.3409 |
| rel. Cp mRNA |  | 0.90 ± 0.32 | 1.00 ± 0.09 | 0.79 ± 0.20 | 1.19 ± 0.39 | 0.94 ± 0.15 | 1.43 ± 0.58 | 1.11 ± 0.17 | 0.92 ± 0.19 | 1.63 ± 0.84 | 1.13 ± 0.14 | 1.09 ± 0.13 | 1.46 ± 0.37 | **0.0053** | **0.0434** | 0.0932 | 0.1214 | 0.7006 | 0.1125 | 0.0573 |
| rel. Ctr1 mRNA |  | 0.96 ± 0.29 | 1.00 ± 0.38 | 0.93 ± 0.16 | 0.92 ± 0.33 | 0.91 ± 0.22 | 0.98 ± 0.29 | 0.97 ± 0.29 | 0.92 ± 0.15 | 0.99 ± 0.36 | 0.91 ± 0.17 | 0.93 ± 0.22 | 1.00 ± 0.22 | 0.8632 | 0.9406 | 0.6965 | 0.8912 | 0.8427 | 0.9056 | 0.8731 |
| rel. Fth1 mRNA |  | 0.99 ± 0.33 | 1.00 ± 0.26 | 1.03 ± 0.26 | 0.97 ± 0.20 | 0.92 ± 0.38 | 1.00 ± 0.37 | 0.96 ± 0.24 | 0.99 ± 0.31 | 0.94 ± 0.20 | 1.14 ± 0.27 | 1.07 ± 0.29 | 1.09 ± 0.19 | 0.9565 | 0.4470 | 0.4282 | 0.8546 | 0.8649 | 0.1410 | 0.9925 |
| rel. Hamp mRNA |  | 0.99 ± 0.22 | 1.00 ± 0.11 | 0.95 ± 0.14 | 1.03 ± 0.21 | 0.99 ± 0.20 | 0.93 ± 0.12 | 1.08 ± 0.19 | 1.38 ± 0.62 | 0.83 ± 0.06 | 0.96 ± 0.17 | 1.01 ± 0.17 | 1.00 ± 0.16 | 0.0724 | 0.5868 | 0.7223 | 0.5136 | 0.2877 | 0.6394 | 0.1535 |
| rel. Heph mRNA |  | 1.02 ± 0.28 | 1.00 ± 0.18 | 0.94 ± 0.22 | 1.04 ± 0.19 | 0.91 ± 0.18 | 0.85 ± 0.15 | 1.02 ± 0.23 | 1.12 ± 0.21 | 0.99 ± 0.16 | 0.91 ± 0.16 | 0.79 ± 0.09 | 0.90 ± 0.18 | 0.2995 | 0.9056 | **0.0066** | 0.5150 | 0.2509 | 0.1455 | 0.4650 |
| rel. Slc40a1 mRNA |  | 1.05 ± 0.24 | 1.00 ± 0.12 | 1.04 ± 0.14 | 1.16 ± 0.20 | 1.03 ± 0.14 | 1.08 ± 0.31 | 1.21 ± 0.17 | 1.18 ± 0.11 | 1.21 ± 0.35 | 1.19 ± 0.20 | 1.09 ± 0.13 | 1.03 ± 0.24 | 0.4030 | 0.0636 | 0.6864 | 0.8820 | 0.5909 | 0.1098 | 0.9243 |
| rel. Slc48a1 mRNA |  | 1.00 ± 0.22 | 1.00 ± 0.22 | 1.07 ± 0.10 | 1.11 ± 0.16 | 1.01 ± 0.13 | 1.10 ± 0.23 | 1.15 ± 0.14 | 1.20 ± 0.16 | 1.12 ± 0.45 | 1.08 ± 0.12 | 1.18 ± 0.27 | 1.16 ± 0.16 | 0.9179 | **0.0352** | 0.6779 | 0.4774 | 0.9329 | 0.4845 | 0.6636 |
| rel. Tfrc mRNA |  | 1.12 ± 0.30 | 1.00 ± 0.07 | 1.09 ± 0.20 | 1.10 ± 0.26 | 0.93 ± 0.19 | 0.86 ± 0.07 | 1.08 ± 0.14 | 1.18 ± 0.52 | 1.00 ± 0.16 | 0.98 ± 0.11 | 1.04 ± 0.11 | 1.01 ± 0.12 | 0.6063 | 0.3395 | **0.0264** | 0.3225 | 0.9966 | 0.5489 | 0.4105 |
| rel. Trf mRNA |  | 0.91 ± 0.27 | 1.00 ± 0.27 | 1.05 ± 0.25 | 0.95 ± 0.21 | 0.98 ± 0.34 | 1.06 ± 0.34 | 1.10 ± 0.37 | 1.14 ± 0.24 | 0.92 ± 0.29 | 1.21 ± 0.28 | 1.08 ± 0.28 | 1.06 ± 0.19 | 0.9376 | 0.1292 | 0.5495 | 0.1382 | 0.6648 | 0.6575 | 0.8500 |
| rel. Trpml1 mRNA |  | 1.14 ± 0.14 | 1.00 ± 0.21 | 1.00 ± 0.07 | 1.11 ± 0.17 | 1.07 ± 0.13 | 1.02 ± 0.13 | 1.14 ± 0.17 | 1.08 ± 0.06 | 0.99 ± 0.19 | 1.08 ± 0.11 | 1.24 ± 0.16 | 1.16 ± 0.10 | 0.1675 | 0.0675 | 0.0846 | 0.1939 | 0.1004 | 0.2705 | 0.4594 |
| rel. Dmt1 mRNA |  | 1.04 ± 0.21 | 1.00 ± 0.12 | 1.15 ± 0.15 | 1.26 ± 0.21 | 1.10 ± 0.13 | 1.08 ± 0.25 | 1.24 ± 0.09 | 1.11 ± 0.18 | 1.07 ± 0.10 | 1.15 ± 0.29 | 1.23 ± 0.23 | 1.14 ± 0.15 | 0.3059 | 0.2226 | 0.1678 | 0.4299 | 0.6126 | 0.5129 | 0.0651 |
| rel. Lrp1 mRNA |  | 1.10 ± 0.19 | 1.00 ± 0.40 | 0.90 ± 0.10 | 1.06 ± 0.25 | 1.12 ± 0.24 | 1.09 ± 0.30 | 1.22 ± 0.26 | 1.03 ± 0.12 | 0.81 ± 0.25 | 1.07 ± 0.33 | 1.02 ± 0.44 | 1.21 ± 0.23 | 0.3516 | 0.7965 | 0.1913 | 0.8011 | 0.0522 | 0.9409 | 0.4622 |
| rel. Lrp2 mRNA |  | 1.54 ± 0.36 | 1.00 ± 0.36 | 1.14 ± 0.33 | 1.35 ± 0.39 | 1.26 ± 0.52 | 0.95 ± 0.26 | 1.00 ± 0.08 | 1.27 ± 0.34 | 1.08 ± 0.42 | 0.94 ± 0.49 | 1.10 ± 0.46 | 0.77 ± 0.15 | 0.0685 | **0.0326** | 0.1936 | **0.0355** | 0.3654 | 0.4033 | 0.3814 |
| rel. Lrp8 mRNA |  | 1.04 ± 0.26 | 1.00 ± 0.33 | 0.84 ± 0.08 | 0.95 ± 0.26 | 1.01 ± 0.38 | 1.07 ± 0.31 | 1.17 ± 0.30 | 1.04 ± 0.23 | 0.95 ± 0.29 | 1.02 ± 0.29 | 1.13 ± 0.29 | 1.16 ± 0.30 | 0.8153 | 0.1405 | 0.3882 | 0.9844 | 0.0898 | 0.9891 | 0.8947 |
| rel. Selenbp1 mRNA |  | 1.21 ± 0.23 | 1.00 ± 0.23 | 1.10 ± 0.11 | 1.18 ± 0.16 | 1.20 ± 0.11 | 1.14 ± 0.19 | 1.01 ± 0.27 | 1.14 ± 0.04 | 1.13 ± 0.12 | 1.07 ± 0.34 | 1.36 ± 0.25 | 1.26 ± 0.25 | 0.6105 | 0.6283 | **0.0317** | **0.0267** | 0.2527 | 0.4753 | 0.9513 |
| rel. Sepp1 mRNA |  | 1.13 ± 0.33 | 1.00 ± 0.03 | 1.10 ± 0.15 | 1.14 ± 0.16 | 1.07 ± 0.22 | 1.04 ± 0.25 | 1.09 ± 0.11 | 1.18 ± 0.34 | 1.00 ± 0.14 | 1.13 ± 0.24 | 1.06 ± 0.13 | 1.12 ± 0.19 | 0.4915 | 0.7497 | 0.8862 | 0.5840 | 0.8857 | 0.9283 | 0.2798 |
| rel. Nis mRNA |  | 0.59 ± 0.27 | 1.00 ± 0.36 | 0.66 ± 0.17 | 1.23 ± 0.60 | 0.70 ± 0.19 | 0.99 ± 0.50 | 1.10 ± 0.56 | 1.10 ± 0.31 | 1.02 ± 0.20 | 0.75 ± 0.13 | 1.09 ± 0.37 | 1.19 ± 0.58 | 0.8455 | **0.0454** | 0.3639 | 0.3713 | 0.1708 | 0.1067 | **0.0128** |
| rel. Oatp1c1 mRNA |  | 1.01 ± 0.27 | 1.00 ± 0.35 | 1.12 ± 0.21 | 0.90 ± 0.25 | 0.92 ± 0.10 | 0.89 ± 0.20 | 0.99 ± 0.19 | 1.03 ± 0.13 | 0.99 ± 0.25 | 0.88 ± 0.27 | 0.92 ± 0.22 | 0.92 ± 0.20 | 0.8530 | 0.7097 | **0.0203** | 0.8702 | 0.8865 | 0.6722 | 0.6926 |
| rel. Mct8 mRNA |  | 0.94 ± 0.29 | 1.00 ± 0.75 | 0.97 ± 0.28 | 1.05 ± 0.46 | 0.87 ± 0.27 | 0.85 ± 0.14 | 0.85 ± 0.15 | 0.93 ± 0.22 | 1.19 ± 0.50 | 0.77 ± 0.33 | 0.89 ± 0.32 | 1.02 ± 0.38 | 0.5475 | 0.9427 | 0.3980 | 0.1616 | 0.7288 | 0.7748 | 0.7946 |
| rel. Lat1 mRNA |  | 1.19 ± 0.24 | 1.00 ± 0.23 | 0.89 ± 0.09 | 1.14 ± 0.25 | 0.99 ± 0.40 | 1.05 ± 0.32 | 1.43 ± 0.29 | 1.12 ± 0.16 | 1.21 ± 0.18 | 1.11 ± 0.46 | 1.35 ± 0.48 | 1.28 ± 0.42 | 0.3728 | **0.0057** | 0.8703 | 0.5691 | 0.1437 | 0.7920 | 0.3740 |
| rel. Lat2 mRNA |  | 1.13 ± 0.45 | 1.00 ± 0.47 | 0.56 ± 0.09 | 0.98 ± 0.33 | 0.86 ± 0.26 | 1.09 ± 0.41 | 1.11 ± 0.36 | 0.88 ± 0.18 | 0.89 ± 0.46 | 1.05 ± 0.59 | 1.30 ± 0.62 | 1.23 ± 0.45 | 0.5657 | 0.1557 | 0.1095 | 0.6408 | 0.0710 | 0.4250 | 0.2919 |
| rel. Slc39a1 mRNA |  | 1.32 ± 0.31 | 1.00 ± 0.16 | 1.00 ± 0.06 | 1.38 ± 0.46 | 1.12 ± 0.20 | 1.31 ± 0.22 | 1.25 ± 0.23 | 1.26 ± 0.21 | 1.27 ± 0.14 | 1.25 ± 0.46 | 1.31 ± 0.27 | 1.24 ± 0.17 | 0.2186 | 0.2564 | 0.1860 | 0.1204 | 0.7743 | 0.2351 | 0.5917 |
| rel. Slc39a2 mRNA |  | 1.10 ± 0.46 | 1.00 ± 0.84 | 0.51 ± 0.15 | 0.88 ± 0.46 | 1.05 ± 0.76 | 0.85 ± 0.65 | 0.99 ± 0.60 | 0.41 ± 0.08 | 0.65 ± 0.32 | 0.94 ± 0.73 | 0.97 ± 0.61 | 1.18 ± 0.62 | 0.5864 | 0.8335 | 0.0955 | 0.2114 | 0.2096 | 0.3331 | 0.7842 |
| rel. Slc39a3 mRNA |  | 1.24 ± 0.21 | 1.00 ± 0.38 | 0.93 ± 0.24 | 1.16 ± 0.31 | 1.13 ± 0.21 | 1.14 ± 0.40 | 1.21 ± 0.32 | 1.11 ± 0.44 | 1.27 ± 0.22 | 1.20 ± 0.30 | 1.37 ± 0.14 | 1.32 ± 0.18 | 0.7871 | **0.0296** | 0.1568 | 0.2444 | 0.2805 | 0.9203 | 0.6705 |
| rel. Slc39a4 mRNA |  | 1.75 ± 0.61 | 1.00 ± 0.80 | 1.99 ± 1.01 | 1.76 ± 0.77 | 1.83 ± 0.83 | 1.73 ± 1.41 | 1.30 ± 0.96 | 2.23 ± 1.47 | 2.08 ± 1.53 | 0.85 ± 0.31 | 1.68 ± 0.98 | 1.61 ± 1.30 | 0.2826 | 0.8338 | 0.5298 | 0.1204 | 0.6887 | 0.1535 | 0.5842 |
| rel. Slc39a5 mRNA |  | 1.12 ± 0.29 | 1.00 ± 0.56 | 0.66 ± 0.13 | 0.89 ± 0.30 | 0.94 ± 0.49 | 1.32 ± 0.89 | 1.23 ± 0.54 | 0.71 ± 0.25 | 1.01 ± 0.43 | 0.97 ± 0.42 | 1.13 ± 0.46 | 1.20 ± 0.71 | 0.7202 | 0.6606 | 0.3076 | 0.8389 | 0.0669 | 0.9789 | 0.2702 |
| rel. Slc39a6 mRNA |  | 1.28 ± 0.18 | 1.00 ± 0.17 | 1.10 ± 0.17 | 1.29 ± 0.29 | 1.10 ± 0.11 | 1.22 ± 0.25 | 1.28 ± 0.15 | 1.29 ± 0.06 | 1.04 ± 0.22 | 1.13 ± 0.29 | 1.27 ± 0.21 | 1.17 ± 0.16 | 0.1065 | 0.4627 | 0.4758 | **0.0124** | 0.1759 | 0.3304 | 0.6925 |
| rel. Slc39a7 mRNA |  | 0.99 ± 0.09 | 1.00 ± 0.13 | 0.97 ± 0.23 | 0.97 ± 0.13 | 1.02 ± 0.11 | 0.96 ± 0.23 | 1.03 ± 0.20 | 0.94 ± 0.16 | 0.98 ± 0.24 | 1.03 ± 0.14 | 1.13 ± 0.16 | 0.97 ± 0.17 | 0.5235 | 0.4829 | 0.4699 | 0.8853 | 0.3656 | 0.3885 | 0.5959 |
| rel. Slc39a8 mRNA |  | 1.13 ± 0.20 | 1.00 ± 0.06 | 1.04 ± 0.02 | 1.12 ± 0.19 | 1.01 ± 0.13 | 1.11 ± 0.25 | 1.19 ± 0.20 | 1.18 ± 0.10 | 1.07 ± 0.22 | 1.14 ± 0.28 | 1.05 ± 0.10 | 1.27 ± 0.18 | 0.2185 | **0.0429** | 0.6949 | 0.7898 | 0.1292 | 0.8895 | 0.4469 |
| rel. Slc39a9 mRNA |  | 1.04 ± 0.19 | 1.00 ± 0.13 | 0.99 ± 0.21 | 1.02 ± 0.17 | 0.90 ± 0.35 | 1.00 ± 0.12 | 1.00 ± 0.12 | 0.81 ± 0.45 | 0.90 ± 0.22 | 1.10 ± 0.14 | 1.04 ± 0.34 | 1.02 ± 0.14 | 0.4633 | 0.1428 | 0.3392 | 0.1326 | 0.8680 | 0.4098 | 0.8304 |
| rel. Slc39a10 mRNA |  | 1.00 ± 0.33 | 1.00 ± 0.22 | 0.98 ± 0.14 | 1.22 ± 0.21 | 1.02 ± 0.11 | 1.06 ± 0.17 | 0.98 ± 0.27 | 1.10 ± 0.08 | 1.02 ± 0.04 | 0.90 ± 0.26 | 0.87 ± 0.32 | 0.99 ± 0.15 | 0.8593 | 0.1410 | 0.9613 | 0.3023 | 0.3010 | **0.0196** | 0.6924 |
| rel. Slc39a11 mRNA |  | 1.19 ± 0.09 | 1.00 ± 0.12 | 1.16 ± 0.14 | 1.15 ± 0.14 | 1.08 ± 0.19 | 0.99 ± 0.19 | 1.07 ± 0.23 | 1.09 ± 0.30 | 1.00 ± 0.14 | 1.07 ± 0.08 | 0.97 ± 0.22 | 1.09 ± 0.22 | 0.191 | 0.2634 | 0.5132 | 0.6333 | 0.9622 | 0.6618 | 0.0813 |
| rel. Slc39a12 mRNA |  | 0.87 ± 0.24 | 1.00 ± 0.06 | 0.87 ± 0.06 | 0.92 ± 0.24 | 0.84 ± 0.14 | 0.80 ± 0.15 | 0.97 ± 0.13 | 1.01 ± 0.34 | 1.03 ± 0.18 | 0.89 ± 0.15 | 0.84 ± 0.17 | 0.79 ± 0.23 | 0.5677 | 0.3526 | **0.0077** | 0.788 | 0.2918 | 0.2263 | 0.7208 |
| rel. Slc39a13 mRNA |  | 0.94 ± 0.28 | 1.00 ± 0.29 | 0.81 ± 0.10 | 0.89 ± 0.21 | 0.97 ± 0.24 | 0.98 ± 0.25 | 1.03 ± 0.24 | 0.92 ± 0.13 | 0.79 ± 0.23 | 0.97 ± 0.21 | 1.02 ± 0.13 | 1.05 ± 0.18 | 0.4849 | 0.5348 | 0.1845 | 0.6499 | 0.0627 | 0.4746 | 0.8458 |
| rel. Slc39a14 mRNA |  | 1.25 ± 0.15 | 1.00 ± 0.30 | 0.96 ± 0.14 | 1.23 ± 0.40 | 1.15 ± 0.30 | 1.25 ± 0.25 | 1.24 ± 0.19 | 1.27 ± 0.13 | 0.92 ± 0.23 | 1.16 ± 0.07 | 1.18 ± 0.11 | 1.19 ± 0.16 | 0.0742 | 0.6823 | 0.0790 | 0.2058 | **0.0226** | 0.3111 | 0.6487 |
| rel. Slc30a1 mRNA |  | 1.14 ± 0.17 | 1.00 ± 0.29 | 0.90 ± 0.10 | 1.13 ± 0.26 | 1.08 ± 0.23 | 1.19 ± 0.16 | 1.28 ± 0.21 | 1.20 ± 0.13 | 0.98 ± 0.14 | 1.11 ± 0.13 | 1.19 ± 0.15 | 1.13 ± 0.19 | 0.0769 | 0.0762 | 0.2048 | 0.3848 | **0.0101** | 0.1330 | 0.9384 |
| rel. Slc30a3 mRNA |  | 0.92 ± 0.48 | 1.00 ± 0.49 | 1.34 ± 0.27 | 0.72 ± 0.32 | 0.73 ± 0.46 | 0.87 ± 0.60 | 0.89 ± 0.49 | 0.92 ± 0.44 | 0.76 ± 0.50 | 0.82 ± 0.61 | 0.88 ± 0.51 | 0.74 ± 0.42 | 0.8626 | 0.3120 | 0.0845 | 0.2753 | 0.9410 | 0.1720 | 0.8817 |
| rel. Slc30a4 mRNA |  | 1.23 ± 0.20 | 1.00 ± 0.34 | 0.85 ± 0.07 | 1.23 ± 0.26 | 1.14 ± 0.50 | 1.31 ± 0.42 | 1.32 ± 0.32 | 1.05 ± 0.20 | 1.03 ± 0.27 | 1.13 ± 0.37 | 1.29 ± 0.30 | 1.24 ± 0.30 | 0.3014 | 0.4596 | **0.0499** | 0.8364 | **0.0443** | 0.4175 | 0.5809 |
| rel. Slc30a5 mRNA |  | 1.03 ± 0.14 | 1.00 ± 0.26 | 0.90 ± 0.02 | 0.99 ± 0.13 | 0.98 ± 0.30 | 0.99 ± 0.18 | 1.09 ± 0.18 | 0.94 ± 0.13 | 1.01 ± 0.12 | 0.93 ± 0.26 | 1.02 ± 0.21 | 1.05 ± 0.11 | 0.8515 | 0.5545 | 0.8993 | 0.5907 | 0.2319 | 0.8257 | 0.5398 |
| rel. Slc30a6 mRNA |  | 1.02 ± 0.15 | 1.00 ± 0.23 | 0.85 ± 0.09 | 1.00 ± 0.24 | 0.92 ± 0.17 | 0.87 ± 0.13 | 1.03 ± 0.22 | 0.94 ± 0.09 | 0.91 ± 0.14 | 1.00 ± 0.20 | 0.98 ± 0.19 | 0.98 ± 0.14 | 0.0634 | 0.4736 | 0.9547 | 0.5986 | 0.7140 | 0.4736 | 0.8322 |
| rel. Slc30a9 mRNA |  | 1.19 ± 0.06 | 1.00 ± 0.19 | 1.12 ± 0.05 | 1.26 ± 0.41 | 1.10 ± 0.10 | 1.05 ± 0.03 | 0.87 ± 0.28 | 1.03 ± 0.32 | 0.91 ± 0.29 | 0.93 ± 0.25 | 1.06 ± 0.28 | 1.06 ± 0.18 | 0.9206 | **0.0093** | 0.2909 | 0.0577 | 0.9796 | 0.6627 | 0.5159 |
| rel. Slc30a10 mRNA |  | 1.14 ± 0.36 | 1.00 ± 0.47 | 0.84 ± 0.13 | 1.20 ± 0.37 | 1.06 ± 0.17 | 0.95 ± 0.07 | 1.06 ± 0.31 | 1.06 ± 0.10 | 0.96 ± 0.19 | 0.73 ± 0.26 | 0.98 ± 0.22 | 0.91 ± 0.08 | 0.1870 | 0.1636 | 0.4969 | 0.0576 | 0.4489 | 0.0511 | 0.6233 |
| rel. 8-oxo-dG level |  | 0.94 ± 0.08 | 1.00 ± 0.07 | 1.07 ± 0.10 | 0.81 ± 0.08 | 1.06 ± 0.10 | 0.95 ± 0.09 | 0.92 ± 0.08 | 0.97 ± 0.08 | 1.15 ± 0.05 | 0.88 ± 0.05 | 0.81 ± 0.02 | 0.95 ± 0.08 | **0.0476** | 0.5495 | **0.0439** | 0.2350 | 0.6488 | 0.4542 | 0.3853 |
| 8-oxo-dG incision | [%] | 36.83 ± 3.09 | 36.51 ± 4.05 | 36.29 ± 3.81 | 37.50 ± 3.35 | 39.72 ± 2.37 | 38.18 ± 3.02 | 29.23 ± 2.37 | 32.02 ± 3.27 | 36.11 ± 3.81 | 37.03 ± 4.26 | 35.70 ± 3.34 | 38.56 ± 2.83 | 0.6552 | 0.1632 | 0.0942 | 0.5906 | 0.9060 | 0.4857 | 0.7144 |
| 5-OH-dU incision | [%] | 20.05 ± 1.30 | 18.49 ± 1.20 | 18.85 ± 2.03 | 16.91 ± 1.66 | 18.17 ± 1.05 | 18.33 ± 1.55 | 17.16 ± 1.36 | 15.33 ± 1.40 | 15.09 ± 1.35 | 18.34 ± 1.82 | 14.13 ± 1.61 | 17.62 ± 1.68 | 0.3558 | **0.0169** | 0.7868 | 0.4254 | 0.6141 | 0.2322 | 0.4776 |
| AP site incision | [%] | 23.97 ± 5.25 | 19.41 ± 2.88 | 23.35 ± 4.77 | 20.98 ± 4.42 | 15.91 ± 2.32 | 20.34 ± 4.36 | 13.50 ± 2.14 | 18.86 ± 3.71 | 13.12 ± 1.53 | 22.20 ± 4.95 | 17.07 ± 2.00 | 21.10 ± 4.20 | 0.6949 | 0.1922 | 0.6960 | 0.6045 | 0.5616 | 0.0799 | 0.6227 |
| mdC/dC | [%] | 3.12 ± 0.03 | 3.23 ± 0.07 | 3.16 ± 0.05 | 3.13 ± 0.04 | 3.19 ± 0.04 | 3.15 ± 0.04 | 3.11 ± 0.04 | 3.17 ± 0.04 | 3.20 ± 0.1 | 3.12 ± 0.04 | 3.12 ± 0.04 | 3.14 ± 0.04 | 0.0939 | 0.4476 | 0.3824 | 0.4313 | 0.5733 | 0.6628 | 0.8728 |
| hmdC/dC | [%] | 0.51 ± 0.03 | 0.56 ± 0.04 | 0.55 ± 0.05 | 0.56 ± 0.03 | 0.53 ± 0.05 | 0.56 ± 0.03 | 0.53 ± 0.05 | 0.51 ± 0.05 | 0.57 ± 0.03 | 0.56 ± 0.04 | 0.57 ± 0.04 | 0.55 ± 0.04 | 0.7719 | 0.9960 | 0.4764 | 0.9745 | 0.6306 | 0.7805 | 0.5007 |
| rel. Atm mRNA |  | 1.23 ± 0.34 | 1.00 ± 0.18 | 1.09 ± 0.41 | 1.18 ± 0.26 | 1.19 ± 0.33 | 1.04 ± 0.41 | 1.12 ± 0.26 | 1.15 ± 0.27 | 1.21 ± 0.20 | 1.26 ± 0.39 | 1.31 ± 0.42 | 1.36 ± 0.39 | 0.9330 | 0.1327 | 0.2355 | 0.4054 | 0.7296 | 0.4317 | 0.7630 |
| rel. Atr mRNA |  | 1.11 ± 0.46 | 1.00 ± 0.36 | 0.98 ± 0.43 | 1.07 ± 0.32 | 1.08 ± 0.41 | 0.84 ± 0.34 | 0.95 ± 0.24 | 1.00 ± 0.35 | 1.13 ± 0.24 | 1.04 ± 0.35 | 1.18 ± 0.39 | 1.22 ± 0.38 | 0.9651 | 0.3503 | 0.5683 | 0.1508 | 0.7061 | 0.3244 | 0.9373 |
| rel. Atrip mRNA |  | 0.99 ± 0.25 | 1.00 ± 0.28 | 0.94 ± 0.31 | 0.87 ± 0.22 | 0.93 ± 0.28 | 0.81 ± 0.30 | 0.98 ± 0.20 | 0.86 ± 0.17 | 0.92 ± 0.14 | 0.85 ± 0.23 | 0.95 ± 0.28 | 0.98 ± 0.20 | 0.9998 | 0.9998 | 0.3427 | 0.5848 | 0.5595 | 0.2784 | 0.6618 |
| rel. Cdkn1a mRNA |  | 1.27 ± 0.88 | 1.00 ± 0.36 | 0.80 ± 0.28 | 0.73 ± 0.20 | 0.91 ± 0.25 | 0.68 ± 0.38 | 0.87 ± 0.24 | 0.82 ± 0.29 | 1.07 ± 0.36 | 0.86 ± 0.28 | 0.84 ± 0.32 | 0.90 ± 0.32 | 0.7793 | 0.9598 | 0.0664 | 0.1013 | 0.5042 | 0.2383 | 0.3151 |
| rel. Chek1 mRNA |  | 1.12 ± 0.20 | 1.00 ± 0.23 | 1.01 ± 0.21 | 1.02 ± 0.20 | 1.07 ± 0.19 | 0.94 ± 0.24 | 0.98 ± 0.15 | 1.12 ± 0.25 | 1.11 ± 0.23 | 1.09 ± 0.17 | 1.06 ± 0.14 | 1.05 ± 0.26 | 0.8406 | 0.3609 | 0.6633 | 0.4413 | 0.7495 | 0.7718 | 0.3286 |
| rel. Chek2 mRNA |  | 1.12 ± 0.19 | 1.00 ± 0.09 | 1.02 ± 0.17 | 1.13 ± 0.15 | 1.17 ± 0.15 | 0.98 ± 0.16 | 0.94 ± 0.13 | 1.03 ± 0.29 | 1.09 ± 0.14 | 0.99 ± 0.10 | 0.96 ± 0.11 | 1.09 ± 0.19 | 0.9821 | 0.1367 | 0.5869 | **0.0114** | 0.6988 | 0.4448 | 0.2006 |
| rel. Mdm2 mRNA |  | 1.06 ± 0.20 | 1.00 ± 0.21 | 0.97 ± 0.18 | 1.00 ± 0.12 | 1.02 ± 0.22 | 0.89 ± 0.28 | 1.00 ± 0.15 | 1.00 ± 0.19 | 1.18 ± 0.26 | 1.01 ± 0.24 | 1.07 ± 0.23 | 1.13 ± 0.21 | 0.8868 | 0.1030 | 0.7120 | 0.0696 | 0.6308 | 0.6255 | 0.9841 |
| rel. Trp53 mRNA |  | 1.05 ± 0.10 | 1.00 ± 0.04 | 1.04 ± 0.10 | 0.98 ± 0.16 | 0.99 ± 0.25 | 1.09 ± 0.12 | 1.06 ± 0.16 | 1.01 ± 0.18 | 0.98 ± 0.07 | 1.08 ± 0.14 | 1.05 ± 0.07 | 1.09 ± 0.15 | 0.5919 | 0.5592 | 0.4969 | 0.5258 | 0.3849 | 0.2851 | 0.9621 |
| rel. Aplf mRNA |  | 1.03 ± 0.08 | 1.00 ± 0.18 | 0.97 ± 0.16 | 1.04 ± 0.13 | 1.07 ± 0.25 | 1.07 ± 0.28 | 1.06 ± 0.13 | 0.96 ± 0.19 | 1.07 ± 0.22 | 1.15 ± 0.27 | 1.08 ± 0.19 | 1.19 ± 0.22 | 0.6473 | 0.2060 | 0.0547 | 0.4873 | 0.8455 | 0.5619 | 0.9405 |
| **Parameter** | **Unit** | **Male** | | | | | | **Female** | | | | | | **Statistics** | | | | | | |
|  |  | **Adult** | | | **Old** | | | **Adult** | | | **Old** | | |  |  |  |  |  |  |  |
|  |  | **-TE** | **+TE** | **+TEaa** | **-TE** | **+TE** | **+TEaa** | **-TE** | **+TE** | **+TEaa** | **-TE** | **+TE** | **+TEaa** | **Diet** | **Sex** | **Age** | **Diet x sex** | **Diet x age** | **Sex x age** | **Diet x sex x age** |
| rel. Aptx mRNA |  | 0.95 ± 0.10 | 1.00 ± 0.04 | 0.94 ± 0.10 | 0.84 ± 0.15 | 0.95 ± 0.21 | 1.01 ± 0.16 | 0.97 ± 0.15 | 0.94 ± 0.14 | 1.00 ± 0.13 | 0.99 ± 0.08 | 0.95 ± 0.06 | 1.01 ± 0.15 | 0.2325 | 0.3074 | 0.8123 | 0.2849 | 0.4812 | 0.4136 | 0.3650 |
| rel. Brca1 mrNA |  | 0.83 ± 0.33 | 1.00 ± 0.40 | 0.80 ± 0.28 | 0.81 ± 0.30 | 0.92 ± 0.53 | 0.75 ± 0.45 | 0.81 ± 0.29 | 0.80 ± 0.36 | 0.97 ± 0.35 | 1.03 ± 0.42 | 0.84 ± 0.27 | 0.76 ± 0.25 | 0.7563 | 0.8605 | 0.8334 | 0.3970 | 0.4870 | 0.6675 | 0.5581 |
| rel. Brca2 mRNA |  | 1.10 ± 0.49 | 1.00 ± 0.37 | 0.90 ± 0.49 | 1.05 ± 0.20 | 1.09 ± 0.45 | 0.89 ± 0.30 | 0.93 ± 0.24 | 0.94 ± 0.36 | 1.07 ± 0.26 | 0.88 ± 0.24 | 1.23 ± 0.38 | 1.09 ± 0.39 | 0.6564 | 0.8060 | 0.5227 | 0.1525 | 0.4479 | 0.6088 | 0.8717 |
| rel. Lig4 mRNA |  | 1.09 ± 0.23 | 1.00 ± 0.11 | 1.00 ± 0.30 | 0.94 ± 0.15 | 1.07 ± 0.22 | 0.95 ± 0.18 | 1.07 ± 0.28 | 1.07 ± 0.21 | 1.17 ± 0.19 | 1.10 ± 0.25 | 1.07 ± 0.24 | 1.07 ± 0.29 | 0.9982 | 0.1013 | 0.5164 | 0.6291 | 0.6323 | 0.8306 | 0.4800 |
| rel. Mre11a mRNA |  | 1.21 ± 0.33 | 1.00 ± 0.15 | 1.04 ± 0.39 | 1.06 ± 0.23 | 1.09 ± 0.32 | 0.97 ± 0.35 | 1.08 ± 0.23 | 1.12 ± 0.23 | 1.18 ± 0.30 | 1.07 ± 0.51 | 1.23 ± 0.39 | 1.21 ± 0.34 | 0.9951 | 0.2370 | 0.9796 | 0.3288 | 0.6076 | 0.5554 | 0.9537 |
| rel. Nbn mRNA |  | 1.04 ± 0.16 | 1.00 ± 0.19 | 0.89 ± 0.07 | 1.01 ± 0.14 | 1.08 ± 0.25 | 1.00 ± 0.27 | 1.01 ± 0.11 | 0.98 ± 0.15 | 1.05 ± 0.06 | 1.09 ± 0.19 | 1.11 ± 0.23 | 1.09 ± 0.15 | 0.7499 | 0.1758 | 0.0870 | 0.3892 | 0.6795 | 0.6705 | 0.6288 |
| rel. Palb2 mRNA |  | 0.97 ± 0.41 | 1.00 ± 0.32 | 0.66 ± 0.19 | 1.00 ± 0.42 | 1.04 ± 0.48 | 1.02 ± 0.44 | 1.05 ± 0.39 | 0.89 ± 0.25 | 1.01 ± 0.25 | 0.94 ± 0.40 | 0.99 ± 0.18 | 1.08 ± 0.32 | 0.8761 | 0.5747 | 0.3039 | 0.3328 | 0.3888 | 0.4359 | 0.6589 |
| rel. Pnkp mRNA |  | 0.99 ± 0.24 | 1.00 ± 0.20 | 0.85 ± 0.09 | 1.07 ± 0.16 | 1.05 ± 0.25 | 1.00 ± 0.25 | 0.96 ± 0.25 | 1.00 ± 0.17 | 1.07 ± 0.11 | 1.07 ± 0.23 | 1.13 ± 0.19 | 1.16 ± 0.18 | 0.9032 | 0.1077 | **0.0282** | 0.1359 | 0.9571 | 0.8444 | 0.7870 |
| rel. Rad50 mRNA |  | 1.16 ± 0.28 | 1.00 ± 0.11 | 1.06 ± 0.31 | 1.09 ± 0.22 | 1.12 ± 0.26 | 1.13 ± 0.17 | 1.09 ± 0.18 | 1.12 ± 0.21 | 1.30 ± 0.24 | 1.19 ± 0.18 | 1.24 ± 0.29 | 1.17 ± 0.22 | 0.7451 | 0.0669 | 0.5233 | 0.5519 | 0.4639 | 0.8825 | 0.3155 |
| rel. Rad51 mRNA |  | 0.97 ± 0.06 | 1.00 ± 0.10 | 0.96 ± 0.07 | 0.99 ± 0.12 | 1.01 ± 0.15 | 0.97 ± 0.26 | 0.93 ± 0.11 | 0.86 ± 0.26 | 1.07 ± 0.15 | 0.98 ± 0.08 | 1.06 ± 0.14 | 1.03 ± 0.09 | 0.5936 | 0.8616 | 0.1805 | 0.1963 | 0.3542 | 0.3336 | 0.3275 |
| rel. Tdp1 mRNA |  | 1.06 ± 0.11 | 1.00 ± 0.20 | 0.99 ± 0.12 | 0.94 ± 0.17 | 1.04 ± 0.23 | 0.99 ± 0.23 | 1.10 ± 0.16 | 1.02 ± 0.14 | 1.10 ± 0.18 | 1.07 ± 0.22 | 1.06 ± 0.15 | 1.09 ± 0.16 | 0.9369 | 0.0834 | 0.7537 | 0.6478 | 0.4998 | 0.7679 | 0.8524 |
| rel. Xrcc4 mRNA |  | 1.01 ± 0.19 | 1.00 ± 0.24 | 0.82 ± 0.07 | 0.95 ± 0.19 | 1.08 ± 0.27 | 1.06 ± 0.24 | 1.10 ± 0.23 | 1.05 ± 0.16 | 1.17 ± 0.16 | 1.04 ± 0.25 | 1.08 ± 0.21 | 1.13 ± 0.19 | 0.8805 | **0.0180** | 0.4876 | 0.2610 | 0.2994 | 0.2306 | 0.4012 |
| rel. Apex1 mRNA |  | 1.00 ± 0.09 | 1.00 ± 0.13 | 0.94 ± 0.13 | 1.00 ± 0.12 | 1.00 ± 0.11 | 0.93 ± 0.14 | 0.96 ± 0.12 | 1.06 ± 0.05 | 1.11 ± 0.12 | 1.00 ± 0.06 | 1.01 ± 0.06 | 0.92 ± 0.07 | 0.3727 | 0.1490 | 0.1339 | 0.2050 | 0.1005 | 0.1580 | 0.1473 |
| rel. Fen1 mRNA |  | 0.92 ± 0.09 | 1.00 ± 0.13 | 0.92 ± 0.17 | 0.96 ± 0.20 | 1.08 ± 0.30 | 1.06 ± 0.27 | 1.01 ± 0.27 | 0.97 ± 0.21 | 1.09 ± 0.20 | 1.03 ± 0.23 | 1.03 ± 0.17 | 1.14 ± 0.25 | 0.4731 | 0.2649 | 0.1604 | 0.3410 | 0.8577 | 0.6294 | 0.9221 |
| rel. Lig1 mRNA |  | 1.12 ± 0.26 | 1.00 ± 0.22 | 0.94 ± 0.45 | 1.17 ± 0.28 | 1.10 ± 0.28 | 0.89 ± 0.40 | 1.01 ± 0.24 | 1.16 ± 0.32 | 1.28 ± 0.10 | 1.09 ± 0.28 | 1.12 ± 0.27 | 1.09 ± 0.38 | 0.8097 | 0.1864 | 0.8703 | 0.0831 | 0.4658 | 0.5058 | 0.8570 |
| rel. Lig3 mRNA |  | 1.10 ± 0.19 | 1.00 ± 0.08 | 1.00 ± 0.23 | 0.97 ± 0.14 | 1.02 ± 0.20 | 1.00 ± 0.31 | 1.14 ± 0.23 | 1.05 ± 0.23 | 1.09 ± 0.19 | 1.07 ± 0.23 | 1.09 ± 0.17 | 1.09 ± 0.21 | 0.8286 | 0.1034 | 0.5774 | 0.9462 | 0.4623 | 0.7806 | 0.9646 |
| rel. Neil1 mRNA |  | 1.06 ± 0.09 | 1.00 ± 0.15 | 1.00 ± 0.10 | 0.97 ± 0.11 | 0.99 ± 0.13 | 0.99 ± 0.17 | 1.01 ± 0.13 | 0.96 ± 0.20 | 1.00 ± 0.18 | 1.02 ± 0.13 | 1.02 ± 0.12 | 1.07 ± 0.14 | 0.8194 | 0.6964 | 0.8820 | 0.8438 | 0.5436 | 0.1416 | 0.9583 |
| rel. Neil2 mRNA |  | 1.16 ± 0.30 | 1.00 ± 0.35 | 1.03 ± 0.51 | 1.25 ± 0.44 | 1.31 ± 0.69 | 1.13 ± 0.61 | 1.09 ± 0.66 | 1.34 ± 0.43 | 1.12 ± 0.41 | 1.33 ± 0.63 | 1.27 ± 0.34 | 1.03 ± 0.60 | 0.4890 | 0.6547 | 0.3847 | 0.8200 | 0.8256 | 0.5305 | 0.6017 |
| rel. Nthl1 mRNA |  | 1.02 ± 0.07 | 1.00 ± 0.08 | 0.99 ± 0.23 | 0.94 ± 0.17 | 1.04 ± 0.13 | 0.92 ± 0.19 | 0.99 ± 0.13 | 0.99 ± 0.07 | 1.10 ± 0.11 | 1.03 ± 0.09 | 1.02 ± 0.06 | 0.98 ± 0.12 | 0.8622 | 0.2266 | 0.3609 | 0.4019 | 0.1549 | 0.7159 | 0.4697 |
| rel. Ogg1 mRNA |  | 0.97 ± 0.11 | 1.00 ± 0.10 | 0.94 ± 0.10 | 0.95 ± 0.13 | 0.90 ± 0.06 | 0.93 ± 0.13 | 0.88 ± 0.11 | 0.90 ± 0.14 | 1.03 ± 0.08 | 0.99 ± 0.05 | 1.01 ± 0.10 | 0.96 ± 0.14 | 0.8478 | 0.5359 | 0.9492 | 0.2654 | 0.3259 | 0.0656 | 0.0640 |
| rel. Parg mRNA |  | 1.05 ± 0.43 | 1.00 ± 0.36 | 0.89 ± 0.36 | 1.01 ± 0.34 | 1.00 ± 0.35 | 0.69 ± 0.34 | 0.80 ± 0.30 | 0.92 ± 0.27 | 1.09 ± 0.08 | 1.04 ± 0.37 | 1.00 ± 0.32 | 0.96 ± 0.43 | 0.6894 | 0.7144 | 0.9144 | 0.1598 | 0.3310 | 0.3440 | 0.8173 |
| rel. Parp1 mRNA |  | 1.12 ± 0.47 | 1.00 ± 0.41 | 0.90 ± 0.43 | 1.07 ± 0.33 | 1.00 ± 0.40 | 0.80 ± 0.40 | 1.02 ± 0.35 | 1.00 ± 0.30 | 1.14 ± 0.31 | 1.08 ± 0.44 | 1.17 ± 0.46 | 1.11 ± 0.48 | 0.7144 | 0.2286 | 0.9451 | 0.3153 | 0.7798 | 0.5059 | 0.9738 |
| rel. Polb mRNA |  | 1.00 ± 0.14 | 1.00 ± 0.16 | 0.88 ± 0.05 | 0.96 ± 0.20 | 1.06 ± 0.27 | 0.98 ± 0.24 | 1.04 ± 0.19 | 0.98 ± 0.15 | 1.04 ± 0.16 | 0.97 ± 0.18 | 1.00 ± 0.22 | 1.03 ± 0.19 | 0.8620 | 0.4577 | 0.7665 | 0.3595 | 0.5280 | 0.4841 | 0.9031 |
| rel. Sirt6 mRNA |  | 1.01 ± 0.21 | 1.00 ± 0.20 | 0.93 ± 0.26 | 1.00 ± 0.21 | 0.90 ± 0.28 | 0.78 ± 0.33 | 0.89 ± 0.17 | 0.96 ± 0.14 | 1.14 ± 0.08 | 0.96 ± 0.24 | 1.04 ± 0.18 | 0.99 ± 0.40 | 0.9790 | 0.2712 | 0.4146 | 0.0881 | 0.3573 | 0.3978 | 0.8106 |
| rel. Xrcc1 mRNA |  | 0.96 ± 0.32 | 1.00 ± 0.19 | 0.78 ± 0.13 | 1.01 ± 0.34 | 1.11 ± 0.44 | 1.10 ± 0.46 | 1.13 ± 0.27 | 1.02 ± 0.22 | 1.11 ± 0.24 | 1.12 ± 0.45 | 1.07 ± 0.36 | 1.37 ± 0.40 | 0.8960 | 0.0664 | 0.0920 | 0.2639 | 0.3044 | 0.6951 | 0.9999 |
| rel. Mgmt mRNA |  | 1.02 ± 0.22 | 1.00 ± 0.21 | 0.94 ± 0.19 | 1.16 ± 0.16 | 1.14 ± 0.25 | 0.94 ± 0.28 | 0.99 ± 0.24 | 1.06 ± 0.30 | 1.22 ± 0.25 | 1.12 ± 0.15 | 1.20 ± 0.22 | 1.23 ± 0.28 | 0.9142 | **0.0424** | 0.0587 | **0.0286** | 0.4410 | 0.9857 | 0.9963 |
| rel. Ercc1 mRNA |  | 0.98 ± 0.12 | 1.00 ± 0.14 | 0.92 ± 0.12 | 1.03 ± 0.12 | 1.00 ± 0.14 | 0.95 ± 0.19 | 0.97 ± 0.15 | 0.96 ± 0.14 | 1.04 ± 0.09 | 1.02 ± 0.12 | 1.05 ± 0.15 | 1.05 ± 0.13 | 0.9499 | 0.2326 | 0.2149 | 0.1905 | 0.8821 | 0.7132 | 0.7542 |
| rel. Ercc4 mRNA |  | 1.00 ± 0.14 | 1.00 ± 0.21 | 0.90 ± 0.19 | 0.96 ± 0.10 | 0.97 ± 0.14 | 0.92 ± 0.11 | 0.93 ± 0.05 | 0.93 ± 0.13 | 1.03 ± 0.06 | 0.93 ± 0.16 | 1.00 ± 0.15 | 0.99 ± 0.20 | 0.8585 | 0.7238 | 0.9381 | 0.1098 | 0.8772 | 0.6729 | 0.6134 |
| rel. Ercc6 mRNA |  | 1.25 ± 0.50 | 1.00 ± 0.51 | 1.04 ± 0.48 | 1.12 ± 0.35 | 1.12 ± 0.42 | 0.83 ± 0.44 | 1.03 ± 0.37 | 1.08 ± 0.33 | 1.31 ± 0.14 | 1.20 ± 0.49 | 1.15 ± 0.39 | 1.13 ± 0.49 | 0.7900 | 0.3296 | 0.7698 | 0.2787 | 0.4166 | 0.6452 | 0.7188 |
| rel. Ercc8 mRNA |  | 1.11 ± 0.18 | 1.00 ± 0.17 | 0.94 ± 0.20 | 0.98 ± 0.14 | 1.01 ± 0.14 | 0.94 ± 0.23 | 1.05 ± 0.14 | 0.99 ± 0.04 | 1.02 ± 0.09 | 0.99 ± 0.18 | 1.05 ± 0.20 | 1.20 ± 0.24 | 0.9261 | 0.1808 | 0.8131 | 0.0929 | 0.1400 | 0.1944 | 0.7516 |
| rel. Xpa mRNA |  | 1.02 ± 0.34 | 1.00 ± 0.27 | 0.91 ± 0.46 | 1.07 ± 0.24 | 1.10 ± 0.42 | 0.95 ± 0.29 | 0.97 ± 0.21 | 0.90 ± 0.22 | 1.10 ± 0.16 | 0.90 ± 0.14 | 1.01 ± 0.16 | 1.10 ± 0.22 | 0.9354 | 0.8550 | 0.5345 | 0.1027 | 0.7283 | 0.7058 | 0.9064 |
| rel. Ctcf mRNA |  | 1.13 ± 0.36 | 1.00 ± 0.43 | 0.77 ± 0.17 | 1.00 ± 0.24 | 1.03 ± 0.38 | 0.87 ± 0.39 | 1.09 ± 0.22 | 0.90 ± 0.12 | 1.10 ± 0.30 | 1.08 ± 0.34 | 1.07 ± 0.35 | 1.09 ± 0.24 | 0.3521 | 0.1981 | 0.7143 | 0.1468 | 0.5616 | 0.7324 | 0.7085 |
| rel. Dnmt1 mRNA |  | 1.29 ± 0.56 | 1.00 ± 0.42 | 1.00 ± 0.54 | 1.16 ± 0.35 | 0.93 ± 0.55 | 0.81 ± 0.35 | 1.01 ± 0.35 | 1.14 ± 0.33 | 1.18 ± 0.41 | 1.18 ± 0.51 | 1.22 ± 0.54 | 1.04 ± 0.47 | 0.4358 | 0.3348 | 0.6324 | 0.2491 | 0.6805 | 0.4106 | 0.8696 |
| rel. Mecp2 mRNA |  | 1.17 ± 0.40 | 1.00 ± 0.23 | 0.94 ± 0.44 | 0.92 ± 0.27 | 0.87 ± 0.33 | 0.80 ± 0.31 | 0.87 ± 0.28 | 0.94 ± 0.33 | 0.87 ± 0.22 | 0.94 ± 0.28 | 0.96 ± 0.35 | 0.93 ± 0.27 | 0.5416 | 0.6287 | 0.3445 | 0.4811 | 0.9518 | 0.0931 | 0.8765 |
| rel. Tdg mRNA |  | 1.26 ± 0.51 | 1.00 ± 0.18 | 0.85 ± 0.26 | 1.14 ± 0.31 | 1.15 ± 0.45 | 0.97 ± 0.42 | 1.21 ± 0.26 | 1.05 ± 0.40 | 1.25 ± 0.31 | 1.10 ± 0.47 | 1.29 ± 0.45 | 1.26 ± 0.41 | 0.6545 | 0.1321 | 0.5823 | 0.1692 | 0.3400 | 0.9637 | 0.9041 |
| rel. Tet1 mRNA |  | 1.16 ± 0.63 | 1.00 ± 0.54 | 0.90 ± 0.62 | 0.94 ± 0.27 | 0.96 ± 0.51 | 0.70 ± 0.39 | 0.99 ± 0.40 | 0.89 ± 0.42 | 0.99 ± 0.34 | 0.93 ± 0.46 | 1.10 ± 0.48 | 1.07 ± 0.44 | 0.7233 | 0.5968 | 0.7064 | 0.3997 | 0.6534 | 0.2469 | 0.9745 |
| rel. Tet2 mRNA |  | 1.27 ± 0.59 | 1.00 ± 0.50 | 0.91 ± 0.58 | 1.05 ± 0.32 | 1.02 ± 0.59 | 0.78 ± 0.39 | 1.06 ± 0.46 | 0.98 ± 0.48 | 1.06 ± 0.29 | 1.07 ± 0.52 | 1.15 ± 0.50 | 1.19 ± 0.50 | 0.5921 | 0.4462 | 0.4462 | 0.3239 | 0.7477 | 0.3201 | 0.9756 |
| rel. Gadd45a mRNA |  | 1.00 ± 0.18 | 1.00 ± 0.26 | 0.97 ± 0.12 | 1.02 ± 0.13 | 1.10 ± 0.21 | 0.96 ± 0.26 | 0.98 ± 0.11 | 0.91 ± 0.27 | 1.11 ± 0.14 | 1.13 ± 0.11 | 1.11 ± 0.21 | 1.17 ± 0.12 | 0.8504 | 0.1230 | **0.0310** | 0.0942 | 0.4078 | 0.1972 | 0.9367 |
| rel. Gadd45g mRNA |  | 1.40 ± 0.47 | 1.00 ± 0.24 | 1.01 ± 0.18 | 1.20 ± 0.29 | 1.18 ± 0.16 | 1.09 ± 0.49 | 1.20 ± 0.35 | 1.11 ± 0.35 | 1.28 ± 0.29 | 1.22 ± 0.35 | 1.49 ± 0.42 | 1.46 ± 0.34 | 0.8035 | 0.0691 | 0.1820 | 0.0729 | 0.1551 | 0.2658 | 0.9392 |
| rel. Bak1 mRNA |  | 1.08 ± 0.36 | 1.00 ± 0.24 | 1.01 ± 0.28 | 1.04 ± 0.24 | 0.98 ± 0.35 | 0.78 ± 0.27 | 0.85 ± 0.26 | 0.90 ± 0.38 | 1.06 ± 0.25 | 0.96 ± 0.32 | 0.98 ± 0.23 | 0.99 ± 0.34 | 0.9486 | 0.7190 | 0.6578 | 0.1974 | 0.4073 | 0.2709 | 0.9777 |
| rel. Bax mRNA |  | 1.11 ± 0.26 | 1.00 ± 0.08 | 1.01 ± 0.34 | 1.06 ± 0.14 | 1.08 ± 0.20 | 0.98 ± 0.24 | 1.09 ± 0.16 | 0.99 ± 0.19 | 1.14 ± 0.17 | 1.06 ± 0.28 | 1.14 ± 0.26 | 1.15 ± 0.22 | 0.8782 | 0.2619 | 0.6108 | 0.3710 | 0.4200 | 0.6186 | 0.9723 |
| rel. Bcl2 mRNA |  | 1.04 ± 0.20 | 1.00 ± 0.08 | 0.88 ± 0.22 | 1.04 ± 0.23 | 0.94 ± 0.11 | 0.81 ± 0.34 | 0.97 ± 0.22 | 0.98 ± 0.04 | 1.09 ± 0.25 | 0.99 ± 0.17 | 1.14 ± 0.28 | 0.99 ± 0.26 | 0.4122 | 0.1275 | 0.8700 | 0.0987 | 0.5335 | 0.4967 | 0.5341 |
| rel. Fas mRNA |  | 1.11 ± 0.59 | 1.00 ± 0.33 | 0.86 ± 0.11 | 0.99 ± 0.17 | 1.03 ± 0.22 | 0.80 ± 0.32 | 0.82 ± 0.11 | 0.78 ± 0.16 | 0.96 ± 0.23 | 1.01 ± 0.16 | 0.95 ± 0.19 | 1.11 ± 0.21 | 0.7209 | 0.6154 | 0.2964 | **0.0192** | 0.8735 | 0.0580 | 0.8342 |
