# Supplementary material for: Long-term suboptimal dietary trace element supply does not affect trace element homeostasis in murine cerebellum
Source: Metallomics. 2024 Jan 31;16(2):mfae003. doi: 10.1093/mtomcs/mfae003 (PMC10873500; doi:10.1093/mtomcs/mfae003)
Supplement: mfae003_Supplemental_Files [file mfae003_supplemental_files.zip › Suppl_data_ Supplementary_Figures.pptx]

## Slide 1
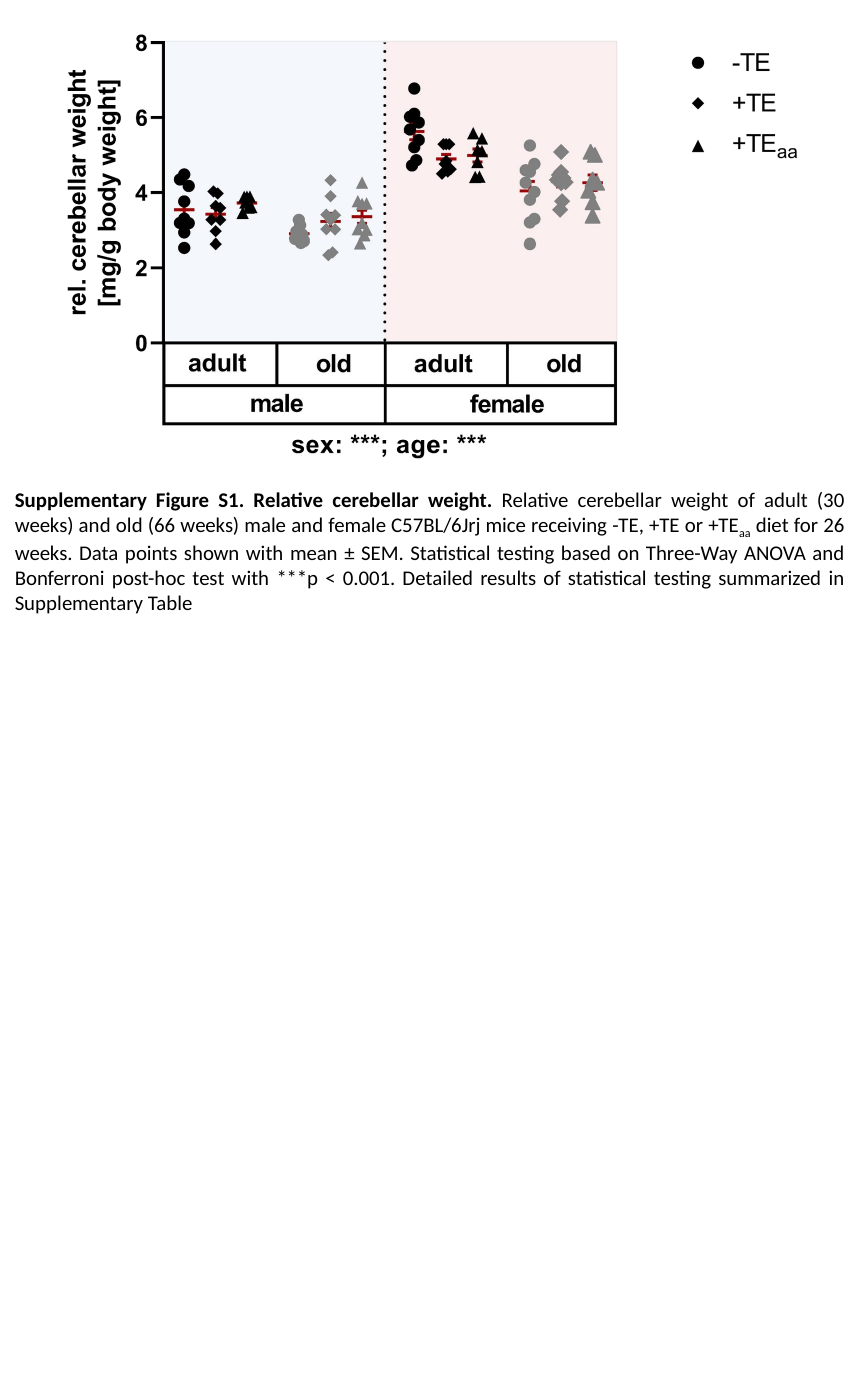

Supplementary Figure S1. Relative cerebellar weight. Relative cerebellar weight of adult (30 weeks) and old (66 weeks) male and female C57BL/6Jrj mice receiving -TE, +TE or +TEaa diet for 26 weeks. Data points shown with mean ± SEM. Statistical testing based on Three-Way ANOVA and Bonferroni post-hoc test with ***p < 0.001. Detailed results of statistical testing summarized in Supplementary Table

## Slide 2
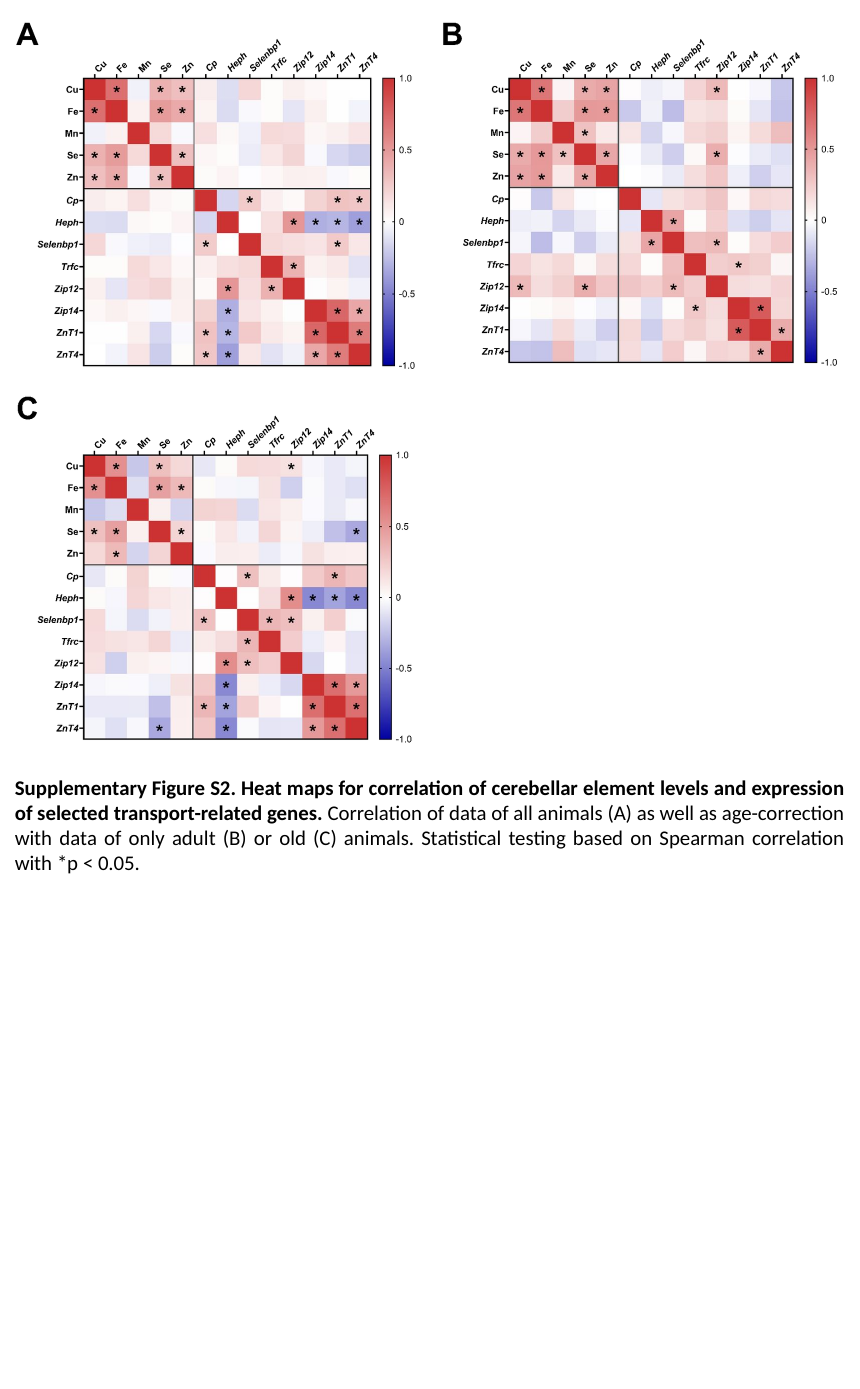

Supplementary Figure S2. Heat maps for correlation of cerebellar element levels and expression of selected transport-related genes. Correlation of data of all animals (A) as well as age-correction with data of only adult (B) or old (C) animals. Statistical testing based on Spearman correlation with *p < 0.05.

## Slide 3
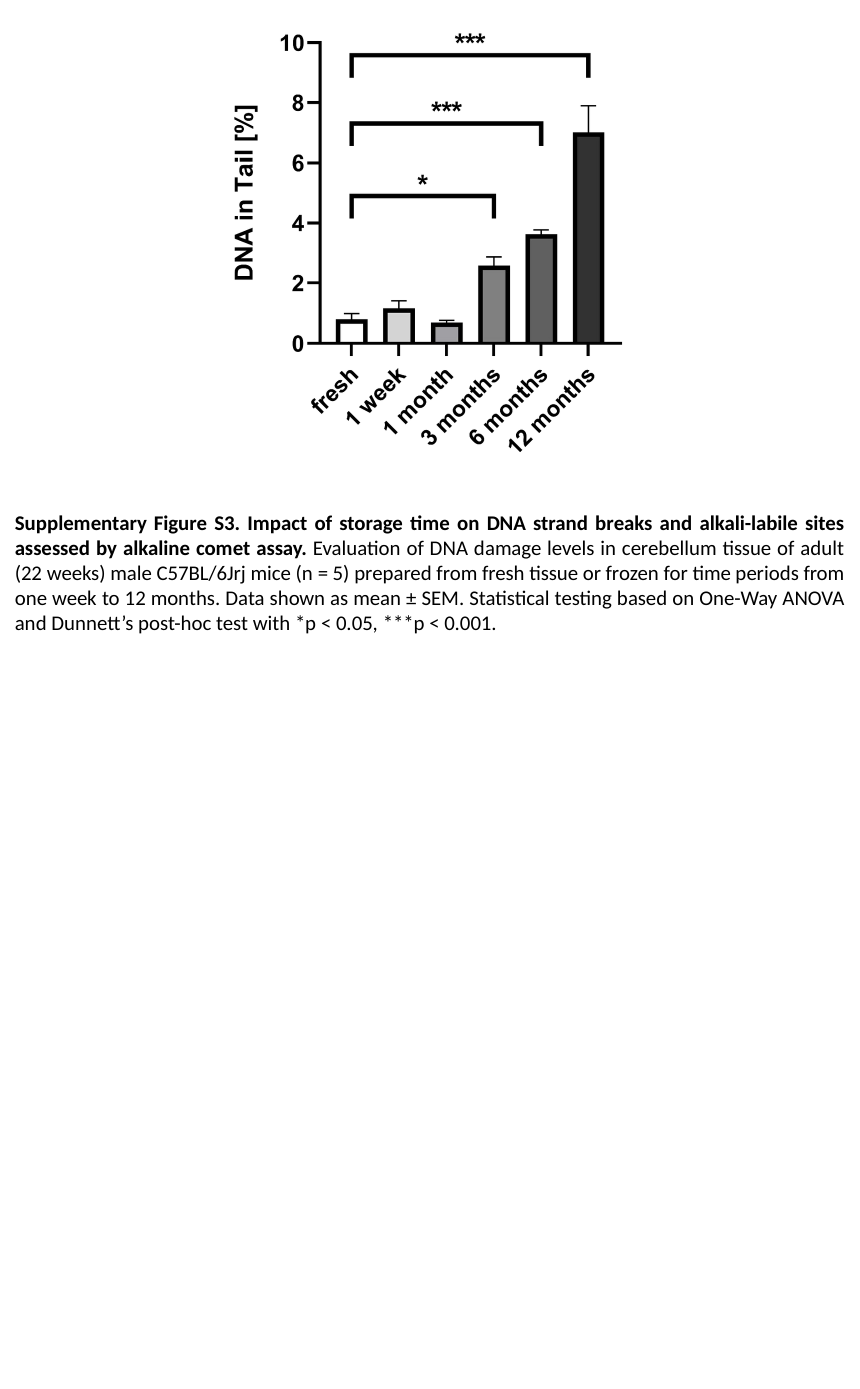

Supplementary Figure S3. Impact of storage time on DNA strand breaks and alkali-labile sites assessed by alkaline comet assay. Evaluation of DNA damage levels in cerebellum tissue of adult (22 weeks) male C57BL/6Jrj mice (n = 5) prepared from fresh tissue or frozen for time periods from one week to 12 months. Data shown as mean ± SEM. Statistical testing based on One-Way ANOVA and Dunnett’s post-hoc test with *p < 0.05, ***p < 0.001.
